# The DAVID Gene Functional Classification Tool: a novel biological module-centric algorithm to functionally analyze large gene lists

**DOI:** 10.1186/gb-2007-8-9-r183

**Published:** 2007-09-04

**Authors:** Da Wei Huang, Brad T Sherman, Qina Tan, Jack R Collins, W Gregory Alvord, Jean Roayaei, Robert Stephens, Michael W Baseler, H Clifford Lane, Richard A Lempicki

**Affiliations:** 1 Laboratory of Immunopathogenesis and Bioinformatics, Clinical Services Program, SAIC-Frederick, Inc., National Cancer Institute at Frederick, Frederick, MD 21702, USA; 2 Advanced Biomedical Computing Center, SAIC-Frederick, Inc., National Cancer Institute at Frederick, Frederick, MD 21702, USA; 3 Computer and Statistical Services, Data Management Services, National Cancer Institute at Frederick, Frederick, MD 21702, USA; 4 Clinical Services Program, SAIC-Frederick, Inc., National Cancer Institute at Frederick, Frederick, MD 21702, USA; 5 Laboratory of Immunoregulation, National Institute of Allergy and Infectious Diseases, National Institutes of Health, Bethesda, MD 20892, USA

## Abstract

The DAVID Gene Functional Classification Tool  uses a novel agglomeration algorithm to condense a list of genes or associated biological terms into organized classes of related genes or biology, called biological modules. This organization is accomplished by mining the complex biological co-occurrences found in multiple sources of functional annotation. It is a powerful method to group functionally related genes and terms into a manageable number of biological modules for efficient interpretation of gene lists in a network context.

## Abstract 2

The DAVID gene functional classification tool uses a novel fuzzy clustering algorithm to condense a list of genes or associated biological terms into organized classes of related genes or biology, called biological modules.

## Rationale

Biological interpretation of large gene lists derived from high-throughput genomic or proteomic studies can be a challenging and daunting process. Some of the difficulties include: acquiring large amounts of functional annotation for every gene; the distributed nature of annotation across numerous sources, that is, not centralized; summarizing which genes are associated with specific biological processes and ranking these processes by over-representation analysis; condensing repetitive or redundant annotation data; identifying functional biological modules consisting of related genes and terms; and viewing inter-relationships between groups of genes and groups of biological terms. A number of publicly available bioinformatics tools have addressed the first three points above, including, but not limited to, GoMiner, DAVID, EASE, GOstat, Onto-express, GoToolBox, FatiGO, GOSSIP, GFINDer, GOBar, and so on [1-25]. The power of many of these applications is to systematically highlight the most over-represented biological terms, out of a list of hundreds or thousands of terms, to increase the likelihood of investigators identifying biological processes most pertinent to the biological phenomena under study [17]. While these tools are extremely useful, they are still weak in mining the many-to-many gene-to-term relationships found in functional annotation databases, as well as in condensing redundant contents.

Individual genes can clearly be associated with multiple biological terms and, conversely, individual biological terms can be associated with multiple genes. These associations form a complex relationship network of 'many-genes-to-many-terms' that represents the true complex nature of biological processes. Data-mining tools that can extract these complex and redundant relationships should be able to identify functional gene-term biological modules. This identification can be accomplished by using exploratory statistical methods that identify groups of genes sharing similar biological terms or, alternatively, identifying groups of biological terms sharing similar genes. For example, if a subset of genes in a list is sodium transporters, then one can expect that they will have major functional annotations in common. A method that can group these genes based on the strength of overlap of the functional annotation should identify modules of related genes and terms. Similarly, terms that have many genes in common can also be grouped into a module of related terms and genes; for example, the terms 'apoptosis', 'cell death', 'death', and 'regulation of cell death' will be grouped together because these terms share a large number of common genes. The advantages of this method of classifying groups of genes and terms into biological modules are: it largely reduces redundant results into a manageable size; it is much easier to understand and visualize gene-to-gene, term-to-term, and gene-to-term relationships, since related genes and terms are brought together in one place; and it is much easier to relate biological modules of interest to a study than it is to relate hundreds of individual terms.

The goals of the project are to identify groups of genes sharing common biology or, alternatively, to identify groups of biological terms sharing common genes relevant to an investigator's study. Most importantly, the heterogeneous annotations/genes can be grouped as long as they are within the same, relevant biological context. In this sense, the definition of functional group in this work is much broader than the traditional concept. The improvement of biological discovery is through better organization of massive and redundant results into a more readable and manageable format (that is biological groups). To this end, we developed the DAVID (The Database for Annotation, Visualization and Integrated Discovery [26]) Gene Functional Classification Tool and the DAVID Functional Annotation Clustering Tool to provide a module-centric approach for functional analysis of large gene lists. First, we developed a new method to measure gene-gene similarity, based on the assumption that genes that share global functional annotation profiles are functionally related to each other. Conversely, we measure term-term similarity based on the assumption that terms that share global gene profiles are functionally related to each other. Then, a DAVID agglomeration method was developed to group related genes or terms into functional groups (biological modules) based on the similarity distances measure. The fuzziness feature of the agglomeration method allows a gene or term to participate in more than one functional group, better reflecting the true 'multiple-roles' nature of genes that can be lost if exclusive methods, such as Hierarchical, K-means, or SOM clustering are used. Functional groups are ranked based on all group members' overall participation in the enriched biological processes associated with the total gene list. A global view of group-to-group relationships is also provided through a unique fuzzy heat map visualization. A subset of 'drill-down' functions associated with each biological module allows investigators to explore and visualize relationships between genes and terms. In this paper, we will mainly describe the key algorithms associated with the DAVID Gene Functional Classification Tool, illustrate the usefulness of several of the functionalities, and demonstrate how quickly investigators can apply the information in a biological module to their study.

## Implementation

**Figure 1:**
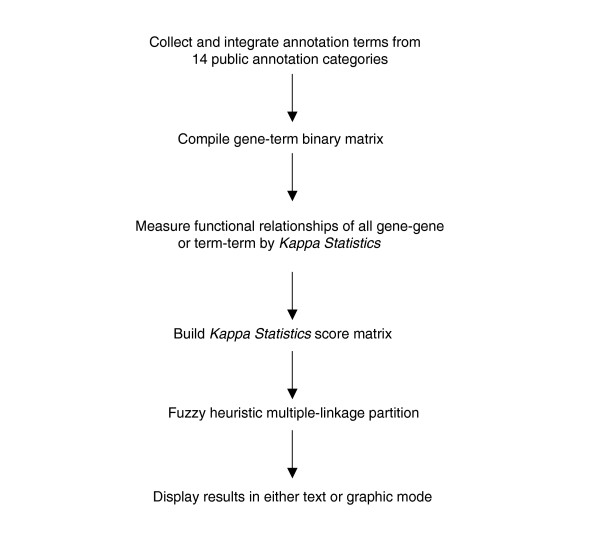
Flow chart of the procedures for the DAVID Gene Functional Classification Tool and the DAVID Functional Annotation Clustering Tool.

The DAVID Gene Functional Classification Tool [27] and DAVID Functional Annotation Clustering Tool [28] are two new components integrated in DAVID Bioinformatics Resources [26]. They were designed as a server-client application on a UNIX server, with the Tomcat web server as the serving engine. Java is the primary language used for calculations as well as the user interface, which utilizes Java Server Page (JSP) technology. In-memory Java data objects containing all mappings between genes and annotation were developed to advance the calculation speed. The DAVID Functional Annotation Clustering Tool uses the same algorithm as the DAVID Gene Functional Classification Tool, but conversely. Therefore, to illustrate the key scientific concepts, we describe only the major procedures of the DAVID Gene Functional Classification Tool. These procedures consist of three major steps: measurement of functional relationship of gene pairs, DAVID agglomeration method to partition genes into functional gene groups, and visualization of results in text and graphic modes (Figure 1).

### Measuring functional relationship of gene pairs based on the similarity of global annotation profiles

The traditional ways of grouping related genes are based on either sequence similarity (sequence homologs), functional categories (protein domain families), or co-expression clusters (microarray clusters). In fact, the majority of co-functioning genes are neither sequence-related nor in the same protein families, such as genes in the same pathway. Therefore, the traditional phylogenetic grouping methods are powerful for evolution-based studies, but too specific and strict to be of much use in classifying genes for the purpose of functional annotation. We propose a novel method to identify related genes by measuring the similarity of their global annotation profiles based on the hypothesis that if two genes have similar annotation profiles, they should be functionally related. This method is able to identify much broader gene groups in which genes share major common biological features as well as tolerate some differences. For example, many different types of genes, with or without too much sequence similarity, could be grouped into a transcription regulation class. We believe that the broader functional groups are more useful for functional annotation purposes and, hence, biological interpretation.

**Figure 2:**
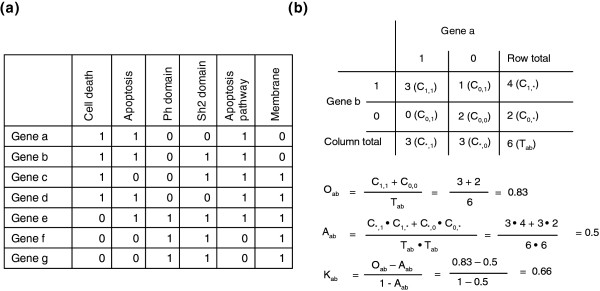
A hypothetical example of detecting gene-gene functional relationships by *kappa *statistics. **(a) **The all-redundant and structured terms are broken into 'independent' terms in a flat linear collection. Each gene associates with some of the annotation term collection so that a gene-annotation matrix can be built in a binary format, where 1 represents a positive match for the particular gene-term and 0 represents the unknown. Thus, each gene has a unique profile of annotation terms represented by a combination of 1 s and 0 s. **(b) **For a particular example of genes *a *and *b*, a contingency table was constructed for *kappa *statistics calculation. The higher *kappa *score (0.66) indicates that genes *a *and *b *are in considerable agreement, more so than by random chance. By flipping the table 90 degrees, the *kappa *score of term-term can be achieved, based on the agreement of common genes (not shown). For more information see Additional data files 11 and 12.

Firstly, a gene-term annotation matrix (Figure 2a) was compiled in a binary mode using thousands of annotation terms in 14 annotation categories (including Gene Ontology (GO), Biological Process, GO Molecular Function, GO Cellular Component, KEGG Pathways, BioCarta Pathways, Swiss-Prot Keywords, BBID Pathways, SMART Domains, NIH Genetic Association DB, UniProt Sequence Features, COG/KOG Ontology, NCBI OMIM, InterPro Domains, and PIR SuperFamily Names) collected in the DAVID knowledgebase [29] (Additional data file 7). Then, *kappa *statistics, a chance-corrected measure of co-occurrence between two sets of categorized data, is adopted to statistically measure the annotation co-occurrence of any given gene pairs [30,31]. Since the annotation profile is in a binary categorical scale, *kappa *statistics is more suitable than the Pearson correlation, which is typically used for continuous, non-categorical data.

For given annotation profiles of genes *m *and *n*, *O*_*mn *_represents the observed co-occurrence, *A*_*mn *_represents chance co-occurrence, and *K*_*mn *_is the *kappa *value representing the degree of annotation co-occurrence between genes *m *and *n*.

$$K_{mn}=\frac{O_{mn}-A_{mn}}{1-A_{mn}}$$

where *K*_*mn *_is 1 for perfect co-occurrence and 0 for co-occurrence no better than random chance (Figure 2b).

**Figure 3:**
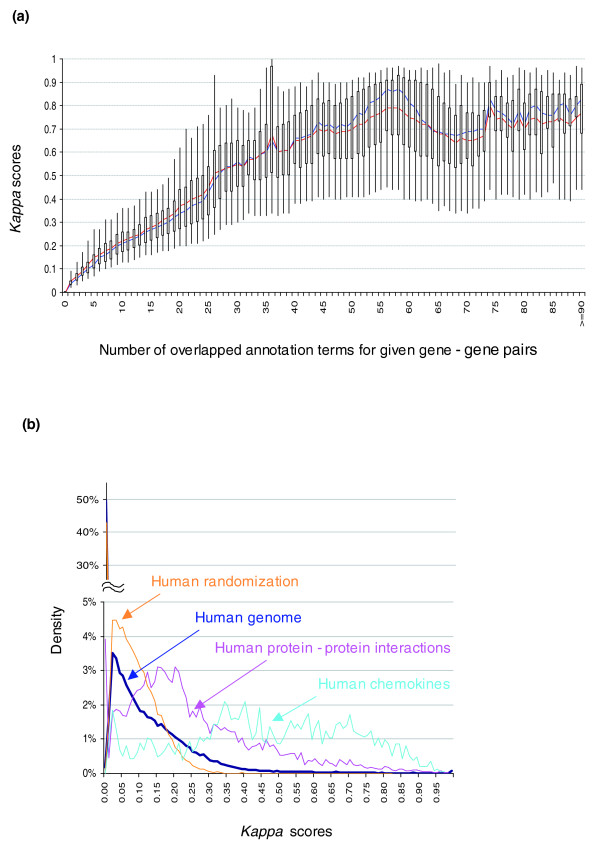
The gene-gene functional relationship can be specifically detected by *kappa *statistics. **(a) ***Kappa *scores were calculated for all possible combinations of human gene-gene pairs (approximately 300 million). Only gene-gene pairs with a higher number of annotation terms in common possibly have good *kappa *values. The box plot consists of the smallest and largest observations at the two end points (95% confidence interval), as well as a box from the 1st to 3rd quartiles. The blue and red lines represent median and mean observations, respectively. **(b) ***Kappa *scores were calculated for all possible human gene-gene pairs, gene-gene pairs with randomized annotation terms, all collected protein-protein interacting pairs, and all 'chemokine' gene pairs, respectively. The distributions of those *kappa *scores from protein-protein interacting pairs (pink) and 'chemokine' gene pairs (light blue) significantly shift to the high value end compared to human total (blue); conversely, the *kappa *score distribution (yellow) of gene pairs with randomized annotation terms remains in the lower value end below 0.35. Interestingly, for the human genome (blue), over 50% of the *kappa *scores equal 0 (no detectable relationships) and >95% are lower than 0.35. Altogether, this indicates that *kappa *statistics can specifically detect the gene-gene functional relationships.

While building the binary annotation matrix, the annotation terms could be redundant or in a structured relationship because many terms from different sources may have the same biological meaning; in addition, GO terms fall into a parent-child relationship in the GO hierarchical structure. Some works demonstrate that gene-term enrichment analysis is improved if the GO hierarchy is considered during the calculation of the enrichment score [32,33]. However, due to the non-hierarchical structure of a majority of annotation sources, we proposed a new 'flat' matrix strategy to break all redundant and structured terms into 'independent' terms in a flat, linear collection (Figure 2a). We believe that an equally weighted, linear, all-inclusive strategy can greatly simplify the situation, as well as maximally leverage the heterogeneous annotations in the similarity measurement (see Additional data files 11 and 12 for more discussion). To answer the question, 'Can this strategy specifically detect the real relationship of gene-gene?', we conducted three studies. The first was to compare the *kappa *score distribution of every possible pair of human genes (approximately 300 million pairs) to that of reported human protein-protein interaction pairs [34]. The protein-protein interaction pairs should have a better chance to co-function in the same biological processes in contrast to random protein-protein pairs. Therefore, a certain degree of functional relatedness should be observed by the method, but not always. If the similarity measurement can specifically detect gene-gene relationships rather than random noise, we would expect to see the score distribution of the protein-protein interaction pairs to shift to the higher value end (Figure 3b). The second study was to detect the *kappa *score distribution of genes specifically selected because their names contained the word 'chemokine'. Since we selected genes with an extreme bias of similarity, we expected the *kappa *scores to give much higher values (Figure 3). The third study was to compare the *kappa *score distribution of all human gene pairs to that of artificial gene pairs with annotation profiles randomly generated, based on the true human annotation frequency. It was expected that the simulated *kappa *scores would be located only in the lower value end (Figure 3). The three independent studies, combined with the extensive test analysis on microarray datasets, strongly supported the strategy that functional similarity measurement is able to specifically detect gene-gene relationships, particularly for the pairs with a *kappa *score 0.35 or above, as suggested by our randomization study (Figure 3; Additional data file 10). However, since the measurement relies on known annotation profiles, this method, like any other high-throughput functional analytical tools, will obviously not work for the genes that lack annotation.

### A novel agglomeration method to classify a gene list into functionally related groups based on the functional similarity scores

After the *kappa *score matrix of all possible pair-wide genes is calculated, it is possible to classify the highly related genes. We examined the typical clustering methods, including hierarchical tree, K-means, hierarchical, FANNY, and SOM. All of them produced weaker clustering results (Additional data file 5) with our test datasets. The poor clustering results stem from one or more of the following weaknesses associated with the aforementioned clustering algorithms. First,: genes must be assigned to one cluster, even though their absolute relationship is weak to all clusters. This results in higher contamination of clusters with noise by forcing membership of weakly related genes. Second, genes can belong to only one cluster, which does not align well with the basic biological nature of genes, in that one gene could participate in multiple, different roles. Third, outliers and uneven cluster sizes can greatly affect clustering quality. Fourth, it is difficult to know the optimal K (number of clusters) for K-means, FANNY, or SOM.

**Figure 4:**
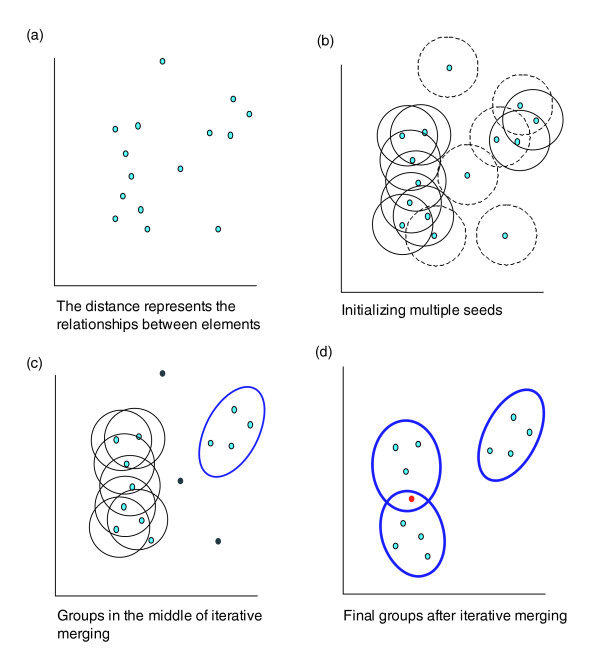
Graphical illustration of the heuristic fuzzy partition algorithm. **(a) **Hypothetically, each element (gene) can be positioned in a virtual two-dimensional space, based on its characteristics (annotation terms). The distance represents the degree of relationship (*kappa *score) among the genes. **(b) **Any gene has a chance as a medoid to form an initial seeding group. Only the initial groups with enough closely related members (for example, members >3 and *kappa *score ≥0.4) are qualified (solid-line circle). Conversely, unqualified ones are shown as dashed-line circles. **(c) **Every qualified initial seeding group is iteratively merged with each other to form a larger group based on the multi-linkage rule, that is, sharing 50% or more of memberships, until all secondary clusters (thicker oval) are stable. Importantly, the genes not covered by any qualified initial seeding group are considered as outliers (in gray). **(d) **Finally, three final groups (thicker ovals) are formed because they can no longer be merged with any other group. One gene (in red) belonging to two groups represents the fuzziness capability of the algorithm. And outliers (in gray in (c)) are removed for clearer presentation. A step-by-step example can be found in Additional data file 13.

This situation motivated us to develop another agglomeration approach, heuristic fuzzy multiple-linkage partitioning, to better reflect the structure of functional annotation data. It can be described as three major steps (Figure 4; see Additional data file 13 for a step-by-step example). Step 1, multiple initial seeds: each gene is selected to serve as a medoid, or center of an initial cluster, as long as it meets minimum relatedness (user input parameters, such as genes, are related to more than three other genes with *kappa *> 0.35) to other genes in the list. Step 2, merge seeds by a minimum, multiple linkage (that is, merge two seeds when they share 50% of their group members). Step 3, repeat step 2 until no more merging can occur.

This method works better than others for this particular type of analysis (Additional data file 5). This method: eliminates overall irrelevant/weak elements, as orphan genes, to significantly push the signal out of noise; allows for fuzziness by allowing genes to be assigned to more than one cluster which aligns with the biological nature; dynamically determines the number of clusters based on the chosen threshold; generates grand groups for easy interpretation; and tolerates outliers extremely well by excluding them in step 1.

There is no gold standard or null hypothesis to evaluate clustering methods and hence no right or wrong answers for any given clustering algorithms. One method may work better than others in the sense that it is more sensitive to the natural structure of a particular problem. However, this method, like any other heuristic approach, has the common weakness that an improper running criteria setting can lead to distorted results. In order to aid less advanced users with the setting of these criteria, we preset five general levels representing combinations of the detailed settings from very low to very high stringencies; based on our extensive tests on multiple datasets, the default stringency level (medium) should be optimal for most cases.

Since there is not a null hypothesis test to compare the quality between clustering algorithms, we try to summarize the quality of our agglomeration algorithm based on randomly selected genes that all clearly belong to one protein family (for example, kinase, phosphatase, chemokine, and so on). Then, the genes were classified by the method. Since we have pre-knowledge about the gene family information, the gene(s) that are grouped incorrectly or excluded from the correct group(s) can be roughly estimated. We observed that the leaking rate (that is, a gene not placed into a group to which it does belong) is between 1% and 2%, and the noise rate (that is, a gene incorrectly placed into a group to which it does not belong) is between 1% and 5%. Most importantly, the method is able to identify key members of groups so that the major biology of each group can quickly be determined. Since the analytical approach is biological module-centric, the major biology associated with each gene group is determined by the majority of gene members rather than by individual genes. Thus, the biology of each group should be very stable, even though there is a chance that a few members are excluded or incorrectly included. In summary, this clustering method shows reasonable performance by eliminating irrelevant, 'noisy' genes and by bringing together strongly related functional groups, while maintaining the fuzzy nature of biology by which genes may be involved in multiple processes.

The last question is, 'Which final functional gene groups are more significant for the experiment?' We extended the traditional enrichment analysis logic so that a gene group is more important if a majority of its gene members is associated with highly enriched annotation terms as found in the traditional enrichment analysis of the total gene list. Thus, the enrichment score of each group is measured by the geometric mean of the EASE Scores (modified Fisher Exact) [2] associated with the enriched annotation terms that belong to this gene group. Importantly, the multiple testing correction issues are considered in the individual EASE scores [2]. And all EASE scores (significant or insignificant) associated with the group participate in the algorithm. In order to emphasize that the geometric mean is a relative score instead of an absolute *p *value, minus log transformation is applied on the geometric mean (Additional data file 6). Therefore, the group enrichment scores are intended to order the relative importance of the gene groups instead of as absolute decision values. A higher score for a group indicates that the group members are involved in more important (enriched) roles. However, all gene groups are potentially interesting despite lower rankings.

### Visualization of results in a very simple text format and a novel fuzzy heat map view

**Figure 5:**
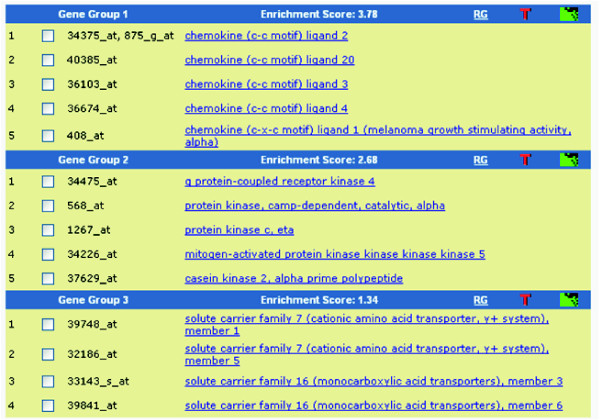
A text format report from the Gene Functional Classification Tool. The example shows the output of 16 genes (Additional data file 1) analyzed by the tool with default settings. Without prior knowledge, the tool is able to classify genes into three functional gene groups. On each group header, a set of buttons is provided for in-depth exploration of the annotation for the group. 'T' reports the major enriched annotation terms associated with the group. The 'Heat Map' symbol provides a detailed graphical view of gene-term relationships. 'RG' searches other related genes in the genome but not in the list.

**Figure 6:**
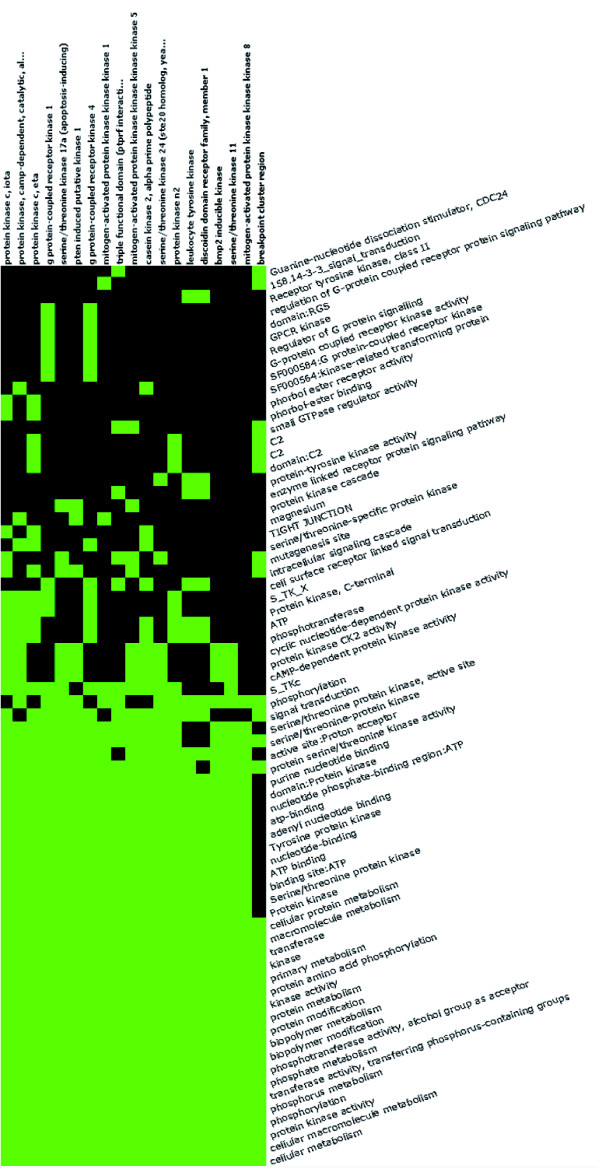
An example of genes-to-terms 2-D view. All the related 23 kinase genes and their associated annotation terms from gene group 3 (kinase group) for demo list 2 are displayed in a 2-D heat map-like interactive graphical view. Green represents the positive association between the gene-term; conversely, black represent an unknown relationship. The annotation terms are ordered based on their enrichment scores associated with the group. The kinase commonly related annotations (big green block) are shown on the left side, and the scattered pattern (green and black) on the right side shows the functional difference.

We implemented both a very simple text format (Figure 5) and a comprehensive novel fuzzy heat map graphic view (Additional data file 4) to present the functional groups derived from the above procedure. The text format simply lists all functional gene groups identified by the algorithm. Although it looks like a linear format, the view allows the user to visualize the multidimensional data of the groups, that is, group members consisting of multiple related genes and terms. Users are able to easily explore the major functional groups by viewing many related genes and annotation terms brought together by the tool. Some accessory, 'drill-down' functionalities (for example, Enriched Terms Report, 2-D View, and so on; Figure 5) are available for each functional group for users to rapidly explore the associated biology in detail. For example, the 'Enriched Term Report' button lists the major annotation terms associated within the functional groups based on the DAVID enrichment engine; the '2-D View' button gives the detailed relationship of genes-to-terms in a two-dimensional heat map view so that the user is able to examine the rich relationship of related genes and annotations in-depth (Figure 6); the 'Related Genes' button allows users to refine the group gene members in different scopes, which can extend the membership of interest and also correct potential type I and II errors in the clustering algorithm (Additional data file 9C). Furthermore, the text format provides links to the list of orphan genes not classified into any functional groups. These genes are orphaned because they do not meet one or more of the partitioning criteria (that is, group membership thresholds, and so on) The list is provided since they may be important genes for the user to examine.

## Results and discussion

We examined the newly developed biological module-centric tools (see Additional data file 8 for a graphical tutorial of using the tools) on two published microarray datasets. It is important to mention that, to avoid potential bias, the datasets of the case studies are different from those used during algorithm development. For the first microarray dataset [35], G1 response genes were identified by microarray experiments after introducing G1 cyclin Cln3p to cln^- ^yeast cells that were previously arrested with cdc34-2. For comparison, the dataset was analyzed by tools with very different algorithms, that is, DAVID Tools [26], GoMiner [16], Ontologizer [33], GOStat [3], ermineJ [36], ADGO [37] and GENECODIS [38]. All tools are able to highlight the major terms (for example, cell cycle, DNA repair, DNA replication, budding, and so on), consistent with previously published observations. However, the DAVID methods are more sensitive to a couple of additional important terms (for example, cyclin-dependant kinase activity, mating, and so on) that were not found among the top terms in the output from the other tools. For more detailed results, comparisons and discussion, see Additional data file 14.

The following detailed discussion is mainly focused on the second microarray dataset [39], of which the gene list is available as demo list 2 on our tool entry page. In this example dataset, authors treated freshly isolated peripheral blood mononuclear cells (PBMCs) with an HIV envelope protein (gp120) and further measured genome-wide gene expression changes using Affymetrix U95A chips [40]. This study provides a global view of the complex interaction between viral and cellular factors, which is an essential mechanism for HIV replication in resting or suboptimally activated PBMCs. A functionally significant annotation of approximately 400 genes (Additional data file 1) derived from the microarray experiment was classified by the authors into five major functional categories: cytokines, chemokines, transcription factors, kinases, and membrane fusion [39]. While the cytokine and chemokine categories were systematically highlighted by EASE (a GO enrichment analysis based on the Fisher Exact Test) [2], other annotation categories reported in the publication were discovered through semi-manual analysis by bioinformatics experts with an advanced level of knowledge of both biology and computer tools.

### The same data re-analyzed by typical functional annotation tools

After the continuous addition of annotations for genes as well as the refinement of gene-term enrichment algorithms during the years since the above study [39] was published, it is interesting to see how the systematic results from current functional annotation tools compare to those reported in this publication. Some of the popular functional annotation tools, such as DAVID Gene Functional Annotation Tool, GOStat, GoMiner, TopGO, Ontologizer, ADGO and GENECODIS [1,3,16,32,33,37,38], were chosen to identify major biological terms with the same gene list. In order to maximally reflect the design spirit of each tool and also make the results more comparable, we kept all default parameters of the tools unchanged, except for synchronizing the data coverage scope within all GO levels (DAVID covers multiple data sources and GOstat covers GO level 3 or above by default). Although all of the testing tools are based on similar gene-term enrichment algorithms, the sensitivity and specificity could be different due to different updates of GO data content, different background gene lists, different score systems, different gene ID mapping schemes, and so on. After obtaining hundreds of annotation terms reported by each of the above tools, the terms, particularly at the top of the results, were compared with each other (Table 1). Approximately 30% of the top terms overlapped between at least two of the tools, for example, cytokine/chemokine activity, inflammatory response, and so on. Some reported terms, for example, kinase, are not ranked at the top by any of the tools (that is GOMiner, 49; DAVID, 24; GOStat, 82; topGO, 76; Ontologizer, 111).

**Table 1:** The top 20 enriched terms for demo list 2 by various traditional functional annotation tools

No.	GOMiner	DAVID Chart	GOstat	Ontologizer	topGO elim	ADGO
1	Inflammatory response	Response to pathogenic bacteria	Cell-cell signaling	Response to stimulus	Induction of positive chemotaxis	Inflammatory response/extracellular region
2	Clathrin coat of coated pit	Chemokine activity	Response to pest, pathogen or parasite	DNA repair	Positive regulation of vascular endothelium	Inflammatory response
3	Viral genome replication	Cell migration	Response to stress	Cell surface receptor linked signal transduction	Chemokine activity	Cell-cell signaling/extracellular space
4	Morphogenesis	Clathrin-coated vesicle	Response to external biotic stimulus	Positive regulation of protein metabolic process	Angiogenesis	Soluble fraction/chemokine activity
5	Cytokine activity	Clathrin vesicle coat	Response to wounding	Cytoskeleton organization and biogenesis	Vascular endothelial growth factor receptor	Extracellular space
6	Establishment of spindle localization	Clathrin coated vesicle membrane	Negative regulation of biological process	Molecular_function	Extracellular matrix binding	Sensory perception/chemokine activity
7	Cell communication	Receptor binding	Negative regulation of physiological process	Cell communication	Viral genome replication	Inflammatory response/chemokine activity
8	Establishment of mitotic spindle localization	Response to other organism	Cytoplasmic vesicle membrane	DNA binding	Extracellular space	Sensory perception/extracellular space
9	Regulation of cellular process	Kinase activity	Cytoplasmic vesicle membrane	Protein binding	Cell-cell signaling	Chemokine activity
10	Regulation of biological process	RNA polymerase II transcription factor activity	Negative regulation of cellular process	Cell cortex	Inflammatory response	Chemotaxis/extracellular space
11	Development	Clathrin coat	Regulation of biological process	Mitochondrial part	Vasculogenesis	G-protein coupled receptor protein signaling pathway/extracellular space
12	Signal transduction	Establishment of cellular localization	Cell proliferation	GTPase activity	Chemotaxis	Inflammatory response/extracellular space
13	Viral infectious cycle	Cell differentiation	Phagocytic vesicle	Chemotaxis	Neutrophil activation	Extracellular space/chemokine activity
14	Positive regulation of protein metabolism	Cell death	Calpain inhibitor activity	Anatomical structure formation	Ammonia ligase activity	G-protein coupled receptor protein signaling pathway/chemokine activity
15	Regulation of protein-nucleus import	Regulation of isotype switching	Cell adhesion	Lyase activity	Endothelin-converting enzyme 1 activity	Chemotaxis/soluble fraction
16	Immune cell migration	Membrane-bound vesicle	Negative regulation of cellular physiological process	Interleukin-12 production	U-plasminogen activator receptor activity	Cell-cell signaling/chemokine activity
17	Organ development	Cell cycle	Vesicle membrane	Nitrogen compound biosynthetic process	Cell adhesion	Cell proliferation/extracellular space
18	Organogenesis	Membrane fraction	Inflammatory response	DNA recombination	Fructose metabolism	Extracellular region/chemokine activity
19	Chemotaxis	Angiogenesis	Cell communication	Cytokine biosynthetic process	Response to pathogenic bacteria	G-protein coupled receptor protein signaling pathway/soluble fraction
20	Taxis	Cell communication	Cell differentiation	Immune system process	Hyaluronic acid binding	Sensory perception/extracellular region
	Total 380 terms (*p *< 0.05)	Total 157 terms (*p *< 0.05)	Total 119 terms (*p *< 0.05)	Total 31 terms (*p *< 0.05)	Total 160 terms (*p *< 0.05)	Total 67 terms (*p *< 0.05)

The example gene list was analyzed by GoMiner, DAVID, GOStat, Ontologizer, topGO, and ADGO. The annotation data coverage was set to GO terms of all levels, and all other parameters used were each tool's default settings. Only the top 20 terms from each tool are shown (see Additional data file 15 for all results). Many of the terms are redundant or found within the same hierarchy. We emphasize the top 20 terms for three reasons: first, the top ranked terms represent the overall quality of the tools in terms of sensitivity and specificity; second, it renders the amount of analytical effects equivalent and comparable throughout the comparisons, including the clustered results; and third, analysts usually spend more time and attention on the top ranked terms due to time and focus constraints.

Even though the results from the tools all point in the same biological direction, there are four obvious problems. First, redundant/similar/hierarchical terms appear in different (significance) positions within the reports (for example, response to stress, response to wounding, response to pathogenic bacteria, response to other organisms, response to external biotic stimulus, inflammatory response, and so on), which makes it difficult for the user to gain or maintain a clear focus of the whole biological picture. It is not easy for users to comprehensively pool all genes related to the same key biology without manually summarizing all related redundant terms. Second, the redundant/similar/hierarchical terms could largely dilute the focus on other key biology that has few or no redundancies (for example, only one term is for establishment of cellular localization). If several redundant/similar/hierarchical terms are represented in the top of the list, less redundant terms may be pushed down the list, possibly decreasing the chance of discovery; for example, a transcription regulation term, reported in an original publication, was not listed in the top 20 by any of the tools. Third, in contrast, due to differences of the annotation levels of different sources, redundant/similar/hierarchical terms may themselves be diluted. While alone a single term may not be at the top of the list, in combination with redundant/similar/hierarchical terms, the biological function may be very significant. Fourth, current tools do not emphasize the inter-relationships between key biological terms (for example, relationships between chemokine/cytokine and signal transduction).

In conclusion, the recent improvement of functional annotation tools provides a powerful means for users to systematically identify key biological functions associated with a gene list. However, due to the weaknesses discussed above, refinement of current gene-term enrichment algorithms and improvement of software usability alone may not address all the issues. Therefore, the development of novel alternative algorithms as a complement is still very necessary.

### The same data analyzed by the DAVID Gene Functional Classification Tool

The same gene list (Additional data file 1) was submitted to our newly developed DAVID Gene Functional Classification Tool described previously (Additional data file 8). The tool is able to efficiently handle up to 3,000 genes at a time, within a few seconds. The tool classified the approximately 400 genes into 16 functional groups (Table 2 and Additional data file 2). The result is much more focused, simplified, and in a manageable size for investigators' interpretation compared to working with a few hundred terms, of which many are redundant in results derived using the traditional tools discussed in the previous section. More importantly, all five reported annotation categories are covered by the 16 functional groups (Table 2). In addition, the tool also lists another 11 interesting gene groups not reported in the original publication. For example, group 13 (tubulin genes) plays a critical role in the nucleation of microtubule assembly. Some studies suggest that HIV infection leads to enteric microtubule depolymerization of infected cells, resulting in increases in HIV permeability [41]. This tool focuses on the overall major common annotation terms associated with a gene group rather than one term or one gene at a time, thereby producing clearer, more concise results that can better allow for focus on the major biology of an experiment. The tool simplifies the results by condensing the redundant terms and summarizing inter-relationships. This analytical logic and presentation format closely mimics how the human brain works and the results better represent the nature of biology.

**Table 2:** Sixteen total gene functional groups identified by the Functional Classification Tool

Gene functional group no.	Associated biology	Group enrichment score
1	Chemokine/cytokine	3.37
2	Transcription regulation	2.89
3	Signal transduction/membrane receptors	2.68
4	Kinase activity	2.54
5	DNA damage/repair	2.23
6	Iron binding	2.05
7	RNA processing/splicing factors	1.81
8	Organic acid transport	1.71
9	Cation/ion transport	1.69
10	DNA metabolism/chromosome organization	1.53
11	Cellular macromolecule catabolism	1.41
12	Metalloprotease	1.34
13	Macrotubule	1.24
14	Protein localization/fusion	1.17
15	Amine metabolism	1.1
16	RAS small GTPase	1.03

The genes of demo list 2 were analyzed by the Functional Classification Tool. The major biology terms associated with each group are manually summarized based on gene-term enrichment buttons provided for each functional group.

The DAVID Gene Functional Classification Tool allows users to further explore a given biological module/gene group in depth. For example, the 'enriched terms' button '2-D View' is able to list all related terms and genes for the kinase group. Thus, a user who is not familiar with kinases can explore the terms of kinase activity, transferase activity, ATP-binding, nucleotide binding, protein metabolism, tyrosine specificity, serine/threonine specificity, regulation of G protein signaling, and signal transduction, and so on in one view at the same time (Figure 6). Therefore, we can quickly learn the biology for the kinase group with the above related terms in a single view and also identify the fine differences among them. For example, there are two G-protein coupling receptor kinases, three protein tyrosine kinases and six kinases involved in cell surface receptor-linked signal transduction among the 23 kinases within the group (Figure 6). The fine details may be very important for pinpointing the key biology associated with a study.

Furthermore, the DAVID Gene Functional Classification Tool allows one gene to be present in more than one functional group, which closely reflects the nature of biology whereby one gene could play multiple roles in different processes. This fuzziness feature improves the chances of discovery by maximally preserving all of the true relationships. For example, *general transcription factor II H *(*GTF2H4/TFIIH, 41371_at*) was assigned to group 2 (transcription regulation group) and group 5 (DNA damage/repair group) (Additional data file 2). Some studies suggest *TFIIH *increases polymerase processivity in HIV infection [42]. Currently, there are few reports about the *TFIIH *DNA repair mechanism being involved in HIV infection, although this DNA repair mechanism could be essential in HIV integration. Hence, the fuzzy capability allows users not only to focus on the *TFIIH *transcription regulation role but also to consider the possible role in HIV integration through the DNA repair mechanism. For another example, *ring finger protein 40 *(*RNF40*) is in group 2 (transcription regulation group) and group 10 (chromosome assembly) (Additional data file 2). Although the biological significance of the ring finger protein in HIV infection is still largely unclear, the annotation result points out two potential areas for further exploration: first, the ring finger protein regulates the tumor necrosis factor-related transcriptional pathway, which is critical to many aspects of HIV transcription; and second, it plays some role in DNA packaging and chromosome integration. Thus, the fuzziness capability is a powerful feature to maximally preserve biological patterns and to discover fine differences for a given gene compared to exclusive methods.

The sensitivity of the Functional Classification Tool can vary with different datasets and stringency criteria. If the running criteria are not suitable to a particular dataset, the output can be distorted. In such cases, some exploration of different running stringencies is necessary in order to obtain the optimal results to meet the expectation of the study.

### The same data analyzed by the Functional Annotation Clustering Tool

Due to the redundancy/hierarchy problems in the results obtained from traditional annotation tools (Table 1), a Functional Annotation Clustering Tool was also developed to organize the highly redundant annotation term results into a simplified and clustered format. This new format allows investigators to focus on an annotation group level by quickly skipping many redundant/similar/hierarchical terms within the group. Compared to 222 individual terms reported by the DAVID Functional Annotation Tool, a traditional term-centric enrichment method, the new tool was able to organize them into 65 annotation clusters (Additional data file 3). For example, the annotation cluster 3 (immune-response group) consists of 11 redundant/similar/hierarchical terms; that is, response to stress, inflammatory response, response to external stimulus, response to pest, pathogen or parasite, and so on. These similar terms are spread throughout the traditional term-centric enrichment report list of 222 terms. Most importantly, the top 20 annotation clusters with a group enrichment score less than or equal to 0.05 (Table 3 and Additional data file 3) contain all annotation categories reported by the original publication, as well as interesting groups not identified. The highly organized and simplified annotation results allow users to quickly focus on the major biology at an annotation cluster level instead of trying to come to the same conclusions by putting together pieces that are scattered throughout a list of hundreds of terms. In addition, the annotation cluster is helpful in comprehensively pooling all related genes associated with an annotation cluster consisting of many related terms. For example, each of the 11 terms within cluster 3 (immune-response cluster) associates with different genes. A pooled gene list brought together by cluster 3 regarding immune-response could be much more comprehensive, compared to the genes selected from one or a few individual terms. Moreover, the tool could possibly bring up the terms not passing the minimum enrichment threshold but highly related to other terms with significant enrichment scores. In conclusion, the clustered result condenses the data into smaller, much more organized biological term modules, which allows investigators to quickly and comprehensively focus on the key biology of interest.

**Table 3:** The top 20 annotation clusters identified by the DAVID Functional Annotation Clustering Tool

Annotation cluster	Representative annotation terms	Enrichment score
1	Negative regulation of biological process	5.38
2	Signal transduction	4.36
3	Inflammatory response	3.75
4	Extracellular region	3.69
5	Cytokine/chemokine activity	3.12
6	Viral genome replication	2.23
7	Cell death/apoptosis	2.19
8	Regulation of biological process	2.18
9	Organ morphogenesis	2.06
10	Regulation of cell cycle	2.01
11	Positive regulation of biological process	1.87
12	Biological process unknown	1.76
13	Physiological interaction between organisms	1.69
14	Antimicrobial humoral response	1.52
15	Transcription cofactor activity	1.46
16	Integral to plasma membrane	1.44
17	Coated vesicle membrane	1.42
18	DNA repair/DNA metabolism	1.38
19	Kinase activity	1.3
20	Myoblast differentiation	1.3

The genes of demo list 2 were analyzed by the Functional Annotation Clustering Tool. The top 20 annotation clusters out of 65 total clusters have group enrichment scores less than or equal to 0.05 (equivalent to 1.3 in minus log scale). The clusters are ordered by group enrichment score. The representative biology terms associated with the top 20 annotation clusters are manually selected, showing a much clearer and non-redundant view of the annotation terms associated with the study.

## Conclusion

The DAVID Gene Functional Classification Tool [26] is able to organize and condense large gene lists into biologically meaningful modules. It changes functional annotation analysis from term- or gene-centric to biological module-centric. This method takes into account the redundant and network nature of biological annotation contents in order to concentrate on the larger biological picture rather than an individual terms or genes. The DAVID Gene Functional Classification Tool is complementary to other functional annotation tools.

## Abbreviations

GO, Gene Ontology; PBMC, peripheral blood mononuclear cell.

## Authors' contributions

DWH oversaw the development of the tool and wrote the manuscript; BTS developed most of the JAVA codes; QT, JC and RS supported some development of functions; GA and JR provided statistical supports; HCL, MWB and RAL supported and supervised the project as principle investigators.

## Additional data files

The following additional data are available with the online version of this paper. Additional data file 1 lists the genes used in the paper. Additional data file 2 provides the complete output in text format for demo list 2 analyzed by the DAVID Gene Functional Classification Tool. Additional data file 3 provides the complete output in text format for demo list 2 analyzed by the DAVID Functional Annotation Clustering Tool. Additional data file 4 is a figure showing the fuzzy heat map visualization of biological modules. Additional data file 5 is a comparison of the novel fuzzy heuristic partitioning method with other clustering methods. Additional data file 6 is an example of the group enrichment score calculation used for the Functional Annotation Clustering Tool. Additional data file 7 describes the fourteen annotation categories used in the DAVID Functional Classification Tool. Additional data file 8 provides graphical instruction and a tutorial on how to use the DAVID Functional Classification Tool and the DAVID Functional Annotation Clustering Tool. Additional data file 9 gives the output examples for the related gene search and related term search. Additional data file 10 is the default setting for minimum overlapped annotation in *kappa *score calculation. Additional data file 11 describes the effect of *Kappa *statistics on biased annotation data. Additional data file 12 provides a hypothetical example to measure the relationships of gene-gene pairs by *kappa *statistics with annotations organized in a 'flat' matrix. Additional data file 13 provides a hypothetical example to demonstrate the general procedure of our agglomeration procedure. Additional data file 14 includes detailed results, comparisons of the new DAVID clustering tools with regards to yeast cell cycle G1 genes [21]. Additional data file 15 gives the annotation results of demo list 2 by GOMiner, GOStat, DAVID chart, topGO, ermineJ ORA, Ontologizer (three methods), ADGO and GENECODIS.

## Supplementary Material

Additional data file 1 Genes used in the paper: 409 Affymetrix IDs of demo list 2; 84 chemokine genes; approximately 17,000 pairs of protein-protein interactions; and 16 Affy IDs. Click here for file

Additional data file 2 Complete output in text format for demo list 2 analyzed by the DAVID Gene Functional Classification Tool. Click here for file

Additional data file 3 Complete output in text format for demo list 2 analyzed by the DAVID Functional Annotation Clustering Tool. Click here for file

Additional data file 4 The genes in demo list 2 were analyzed by DAVID Gene Functional Classification Tool. The identified biological groups/modules were displayed by the fuzzy heat map. Click here for file

Additional data file 5 The binary gene-term matrix (like Figure 2a) was compiled and submitted to different clustering engines, including Hierarchical clustering, and K-means. The results were evaluated and compared side-by-side. Click here for file

Additional data file 6 An example of the group enrichment score calculation used for the Functional Annotation Clustering Tool. Click here for file

Additional data file 7 Fourteen annotation categories used in the DAVID Functional Classification Tool. Click here for file

Additional data file 8 Graphical instruction and tutorial on how to use the DAVID Functional Classification Tool and the DAVID Functional Annotation Clustering Tool. Click here for file

Additional data file 9 **(a) **Related gene search for 'interleukin 8' in the scope of demo list 2. **(b) **Related term search for 'inflammatory response' in the scope of all annotations. **(c) **Related gene search for a group of genes, group 1 for demo list 2, identified by the DAVID Gene Functional Classification Tool. Click here for file

Additional data file 10 **(a) **Significant *kappa *scores (≥0.35 based on randomization study in Figure 3) can be obtained only for gene-gene pairs with higher overlapped annotation terms (≥10). Thus, there is no reason to calculate *kappa *scores, in an attempt to save the calculating time for DAVID Functional Classification, for the large number of those gene-gene pairs with fewer annotation terms overlapped. A conservative default filter is 4. **(b) **Such a default filer (blue curve) has somewhat greater impact on the significant *kappa *scores in the higher end, compared to those in the lower end. However, it will skip a significant amount of *kappa *calculation of gene-gene pairs. Click here for file

Additional data file 11 The annotation data contents contain many more 0 s than 1 s. The test shows that *kappa *statistics is able to detect 1-1 relationships, which are the key biological co-occurrences that we desire to measure. Click here for file

Additional data file 12 The examples suggest that the 'flat' matrix strategy, along with *kappa *statistics, allows for the quantitative measurement of gene-gene and term-term relationships based on global annotation profiles. All levels of annotation are important to measurement contribution. Click here for file

Additional data file 13 The example provides a step-by-step demonstration of the clustering algorithm, thereby showing how the members are grouped together, how the number of total groups are determined, and how fuzziness can occur. Click here for file

Additional data file 14 Detailed results, comparisons and discussion of the new DAVID clustering tools with regards to yeast cell cycle G1 genes [21] Click here for file

Additional data file 15 The annotation results all point in the right direction, that is, inflammatory responses. However, the redundant/similar/hierarchical terms are spread throughout the results, which decreases analytical efficiency. In addition, some of the key terms reported by the original publication are not on the top of the results produced by other tools, but are always covered by the DAVID tools. Click here for file
