# Supplementary material for: The DAVID Gene Functional Classification Tool: a novel biological module-centric algorithm to functionally analyze large gene lists
Source: Genome Biol. 2007 Sep 4;8(9):R183. doi: 10.1186/gb-2007-8-9-r183 (PMC2375021; doi:10.1186/gb-2007-8-9-r183)
Supplement: Additional data file 4 — The genes in demo list 2 were analyzed by DAVID Gene Functional Classification Tool. The identified biological groups/modules were displayed by the fuzzy heat map. [file gb-2007-8-9-r183-S4.ppt]

## Slide 1
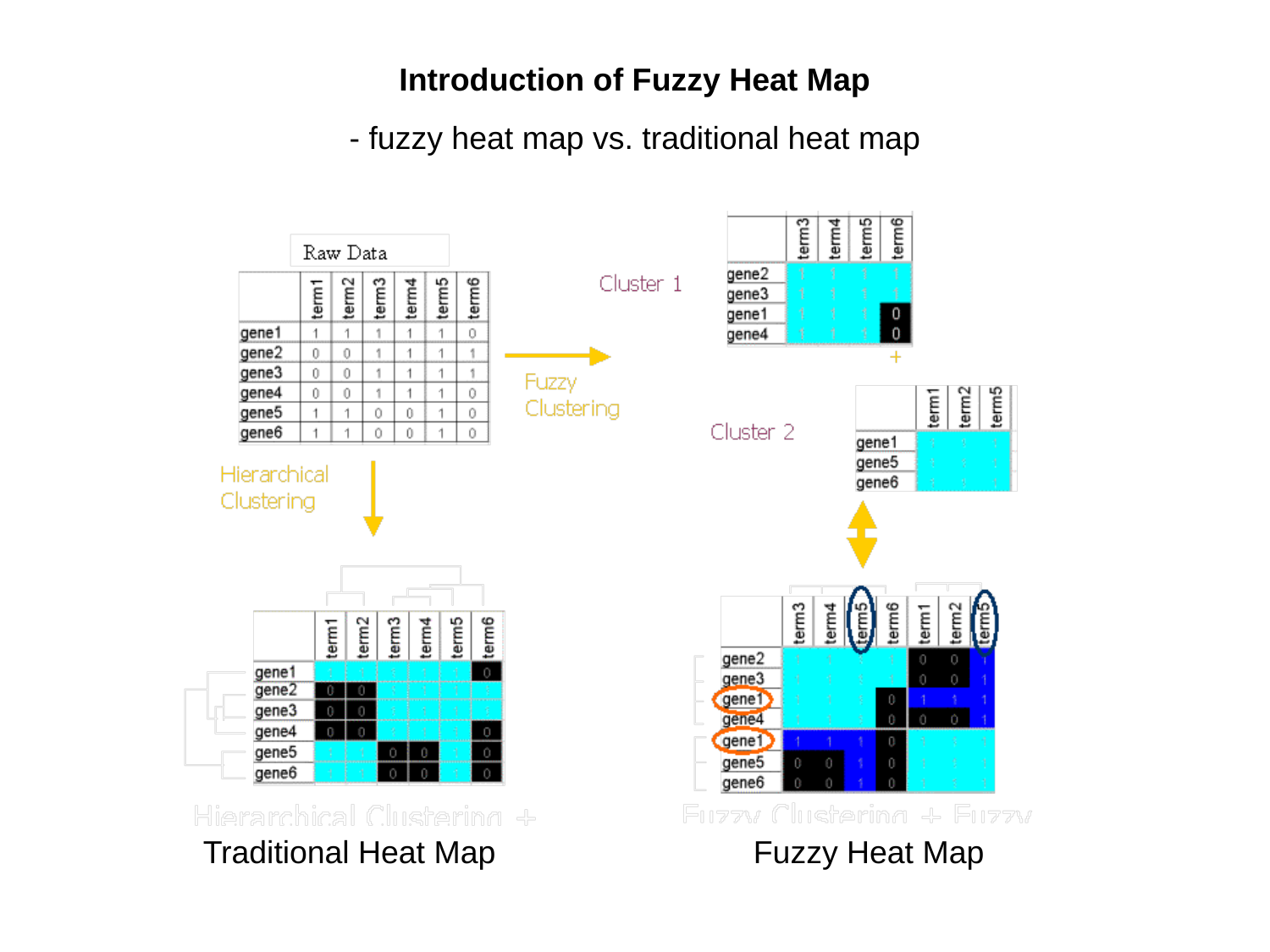

Introduction of Fuzzy Heat Map
- fuzzy heat map vs. traditional heat map
Traditional Heat Map
Fuzzy Heat Map

## Slide 2
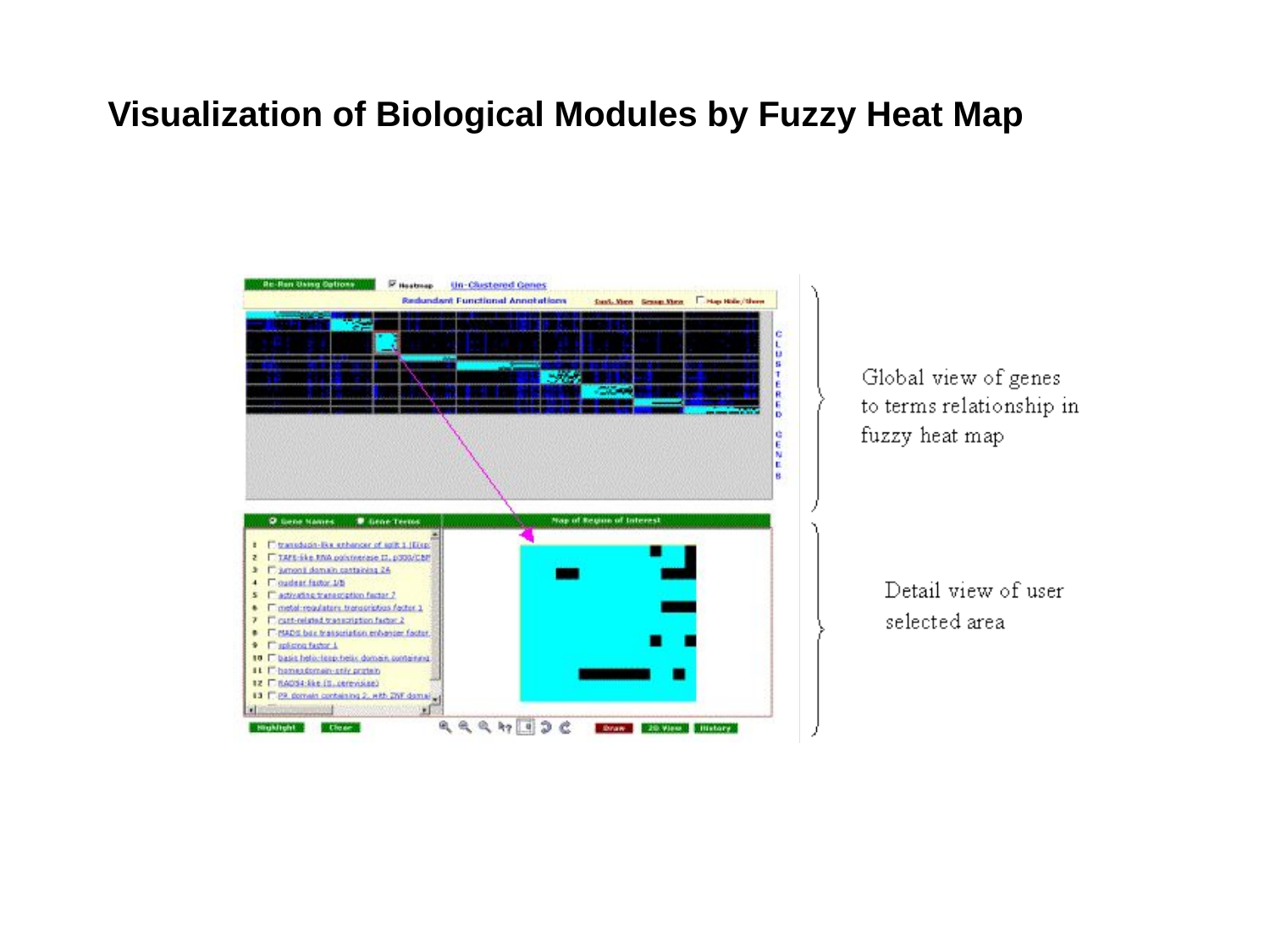

Visualization of Biological Modules by Fuzzy Heat Map

## Slide 3
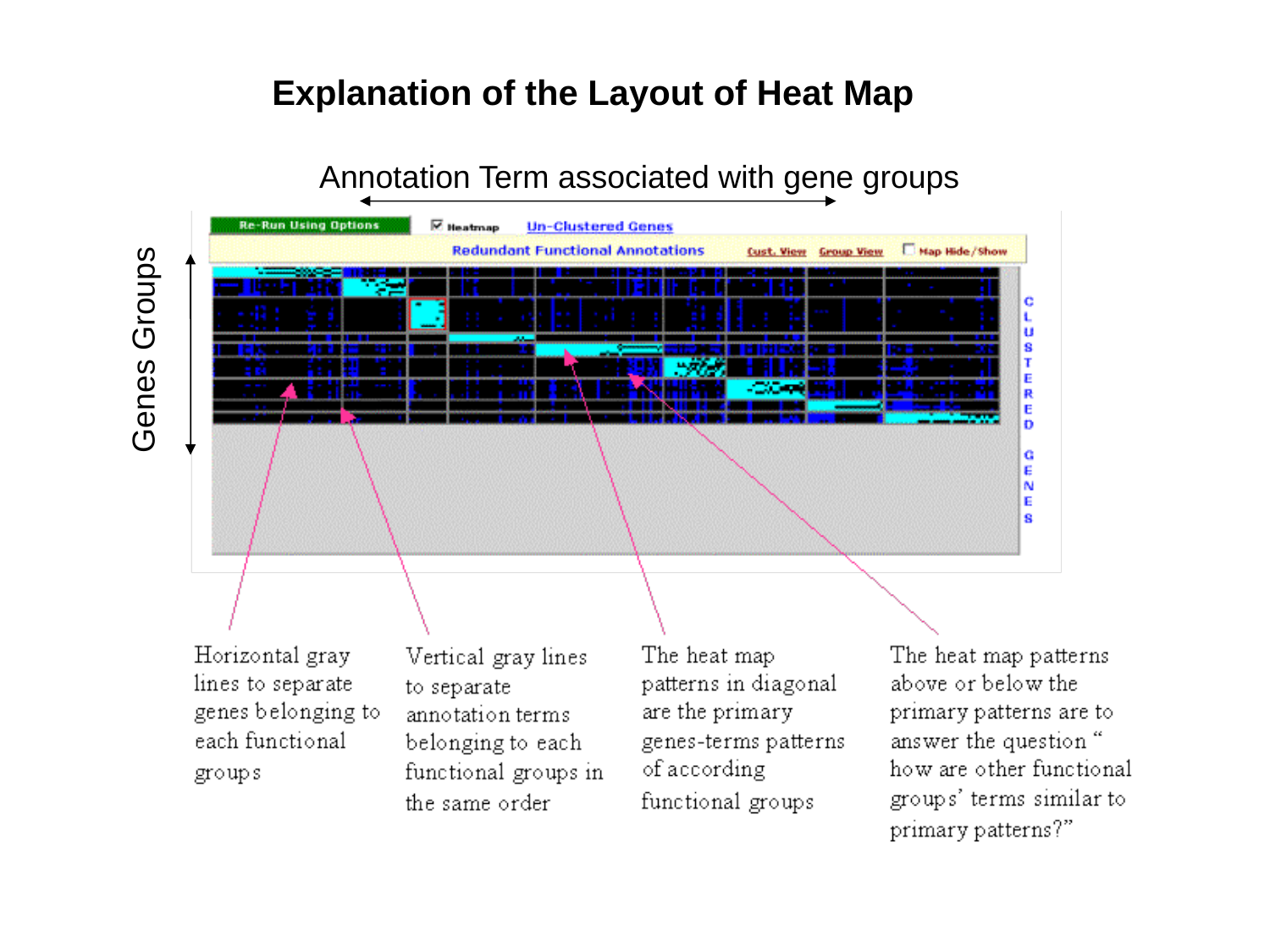

Explanation of the Layout of Heat Map
Annotation Term associated with gene groups
Genes Groups

## Slide 4
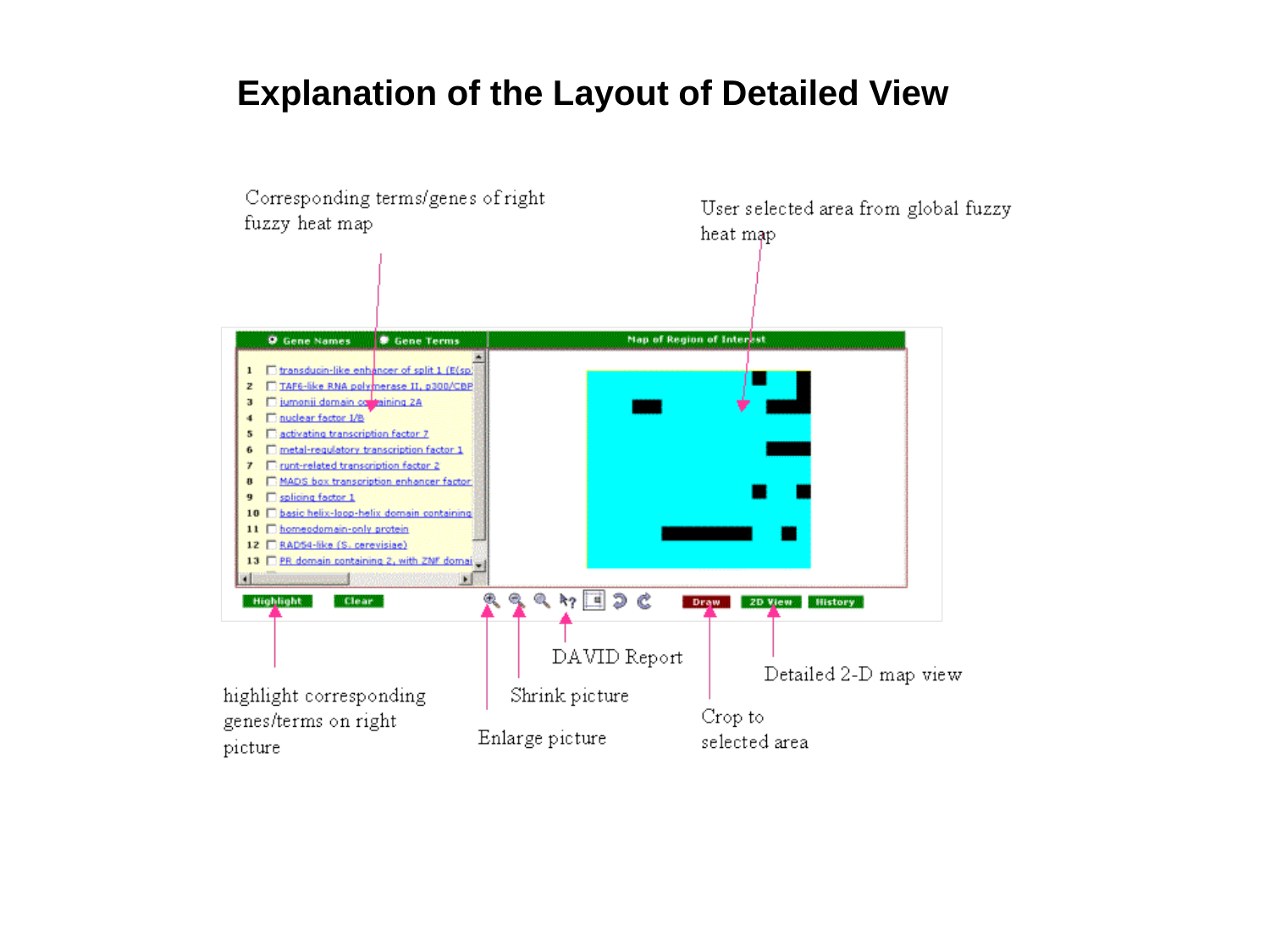

Explanation of the Layout of Detailed View
