# Supplementary material for: The DAVID Gene Functional Classification Tool: a novel biological module-centric algorithm to functionally analyze large gene lists
Source: Genome Biol. 2007 Sep 4;8(9):R183. doi: 10.1186/gb-2007-8-9-r183 (PMC2375021; doi:10.1186/gb-2007-8-9-r183)
Supplement: Additional data file 5 — The binary gene-term matrix (like Figure 2a) was compiled and submitted to different clustering engines, including Hierarchical clustering, and K-means. The results were evaluated and compared side-by-side. [file gb-2007-8-9-r183-S5.doc]

**Side-by-side comparison of DAVID Agglomeration, K-medoids (PAM) and Hierarchical clustering methods with the same HIV gene list**

**I. The study design and overview**


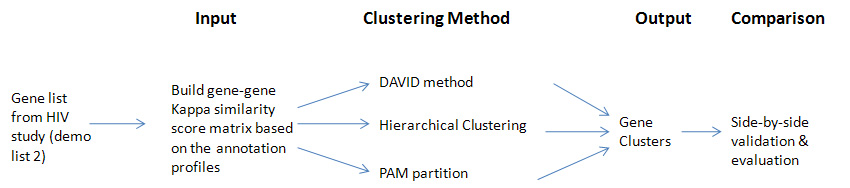


**II. Procedure and results**

**Step 1. Get gene list (Cicala C., et al., ref 39) and build gene-gene Kappa similarity score matrix as the way described in the manuscript.**

**Step 2. Run DAVID, K-medoids (PAM), Hierarchical Clustering methods with above matrix**

**2.1** Determine the # of final gene clusters

| **DAVID Agglomeration** | **PAM** | **Hierarchical Clustering** |
| --- | --- | --- |
| Systematically determined by the algorithm itself. | Since we have pre-knowledge that the HIV gene list has at least 16 major gene classes in our previous studies. We chose 25 for K values. Hopefully, PAM method can generate ~16 very good gene clusters with some other 'ill-defined' clusters. The 'ill-defined' clusters can be discarded thereafter. | For the same reasons of PAM, 25 gene clusters were obtained based on the dendrogram for fair comparison. |

**2.2** Run each of the clustering methods

DAVID Clustering method was ran on DAVID website; Others were tested with R.

**Step 3. Validation and evaluation of the output gene clusters from three methods**

**3.1** how the members in each cluster are compact?

| 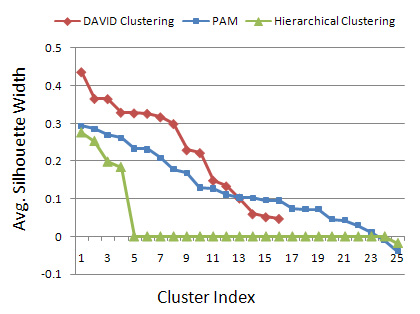 | 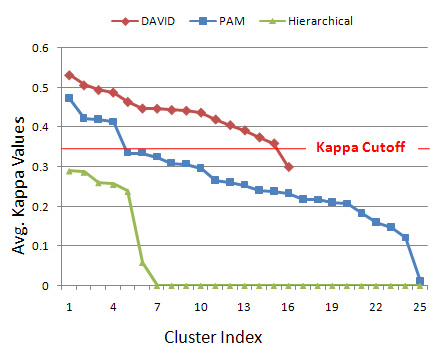 |
| --- | --- |
| The avg. silhouette width/values for each of gene clusters were calculated. The clusters were ordered by the sil. values from large to small. The clusters generated by DAVID show higher sil. values. Most of the clusters by PAM and HC in lower sil. values instead of only a few. It indicates that majority of the PAM and HC clusters are not’ healthy’. | The avg. Kappa values for each of gene clusters were calculated. The clusters were ordered by Kappa values from large to small. The clusters generated by DAVID show much higher avg. Kappa values, most of which are above Kappa cutoff 0.35 (suggested by fig. 3). Most avg. Kappa values of clusters obtained from PAM and HC are below 0.35. It indicates the quality of most of the PAM and HC clusters are impacted by the biased input data, i.e. becoming 'ill-defined' clusters. |

**3.2** how stable are clustering results?

| 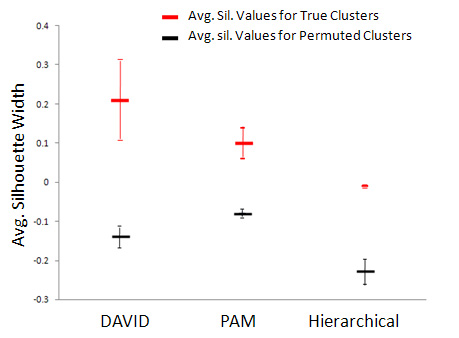 | 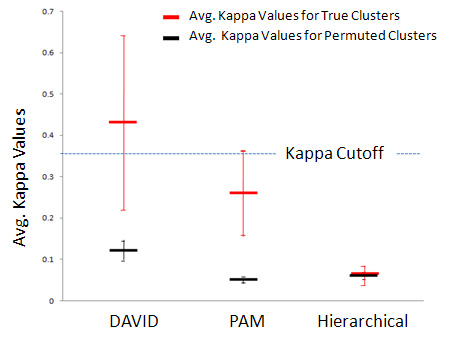 |
| --- | --- |
| The members in the true clusters were permuted for 200 times. The avg. sil. values were calculated for each permuted clusters. The true DAVID clusters have the highest avg. sil. values, as well as are most distanced from permuted clusters. | The members in the true clusters were permuted for 200 times. The avg. Kappa values were calculated for each permuted clusters. The true DAVID clusters have the highest avg. Kappa values, as well as are most distanced from permuted clusters. In addition, the clusters of PAM and HC are all below Kappa cutoff 0.35. |

**3.3** how are cluster members biologically relevant?

A n example: comparison of membership for chemokine cluster generated by DAVID, PAM and HC.

| **DAVID Cluster 1** | **PAM Cluster 7** | **Hierarchical Clustering ??** |
| --- | --- | --- |
| 1. chemokine (c-c motif) ligand 2 2. chemokine (c-c motif) ligand 20 3. chemokine (c-c motif) ligand 3 4. chemokine (c-c motif) ligand 4 5. chemokine (c-x-c motif) ligand 1 (melanoma growth stimulating activity, alpha) 6. interleukin 8 | 1. chemokine (c-c motif) ligand 2 2. chemokine (c-c motif) ligand 20 3. chemokine (c-c motif) ligand 3 4. chemokine (c-c motif) ligand 4 5. chemokine (c-x-c motif) ligand 1 (melanoma growth stimulating activity, alpha) 6. interleukin 8 7. **chorionic somatomammotropin hormone 1 (placental lactogen)** 8. **defensin, beta 4** 9. **deleted in malignant brain tumors 1** 10. **serpin peptidase inhibitor, clade a (alpha-1 antiproteinase, antitrypsin), member 3** 11. **transforming growth factor, beta-induced, 68kda** 12. **tumor necrosis factor, alpha-induced protein 6** 13. **charcot-leyden crystal protein** | Failed |

Evidence 2-D view for PAM Cluster 7


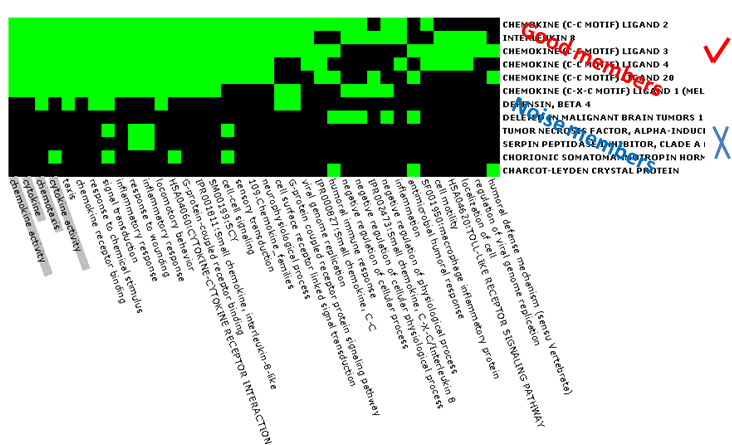


The genes (# 7 - # 13) highlighted in blue do not have any terms related to ‘chemokine activity’. Thus, they are not appropriate to be included in the chemokine group by PAM. The example explains that most, if not all, of the clusters can easily get contamination (i.e. becoming 'ill-defined' clusters) due to the less tolerance of biased input data by the PAM and HC methods. DAVID method is able to agglomerate them in a more accurate way.

**III Conclusion**

**Since most of the clustering methods take into consideration all members during the procedure, the biased and sparse dataset with many outliers, like the gene-annotation matrix in this project, can largely disturb the WHOLE clustering procedure instead of only one or a few clusters. As a result, most of, if not all clusters, in the output, are not helpful (i.e 'ill-defined' clusters). In the end, users do not have a chance to discard a few 'ill-defined' clusters and collect good ones, because all of the clusters are 'ill'. Therefore, typical clustering methods shown in this study will generate many good clusters with a few 'ill-defined' ones only on the dataset that contains tighter membership with only a few outliers. The very biased and sparse dataset, such as the gene-annotation matrix, is obviously not the case. In addition, consistent good performances on multiple real experimental datasets described in the manuscript are further supporting evidences of our clustering method. However, ours does not decrease the value of other methods because all clustering methods have their unique values for different situations or data types. Thus, our conclusion is that our clustering procedure is working well for this particular situation.**
