# Supplementary material for: The DAVID Gene Functional Classification Tool: a novel biological module-centric algorithm to functionally analyze large gene lists
Source: Genome Biol. 2007 Sep 4;8(9):R183. doi: 10.1186/gb-2007-8-9-r183 (PMC2375021; doi:10.1186/gb-2007-8-9-r183)
Supplement: Additional data file 6 — An example of the group enrichment score calculation used for the Functional Annotation Clustering Tool. [file gb-2007-8-9-r183-S6.ppt]

## Slide 1
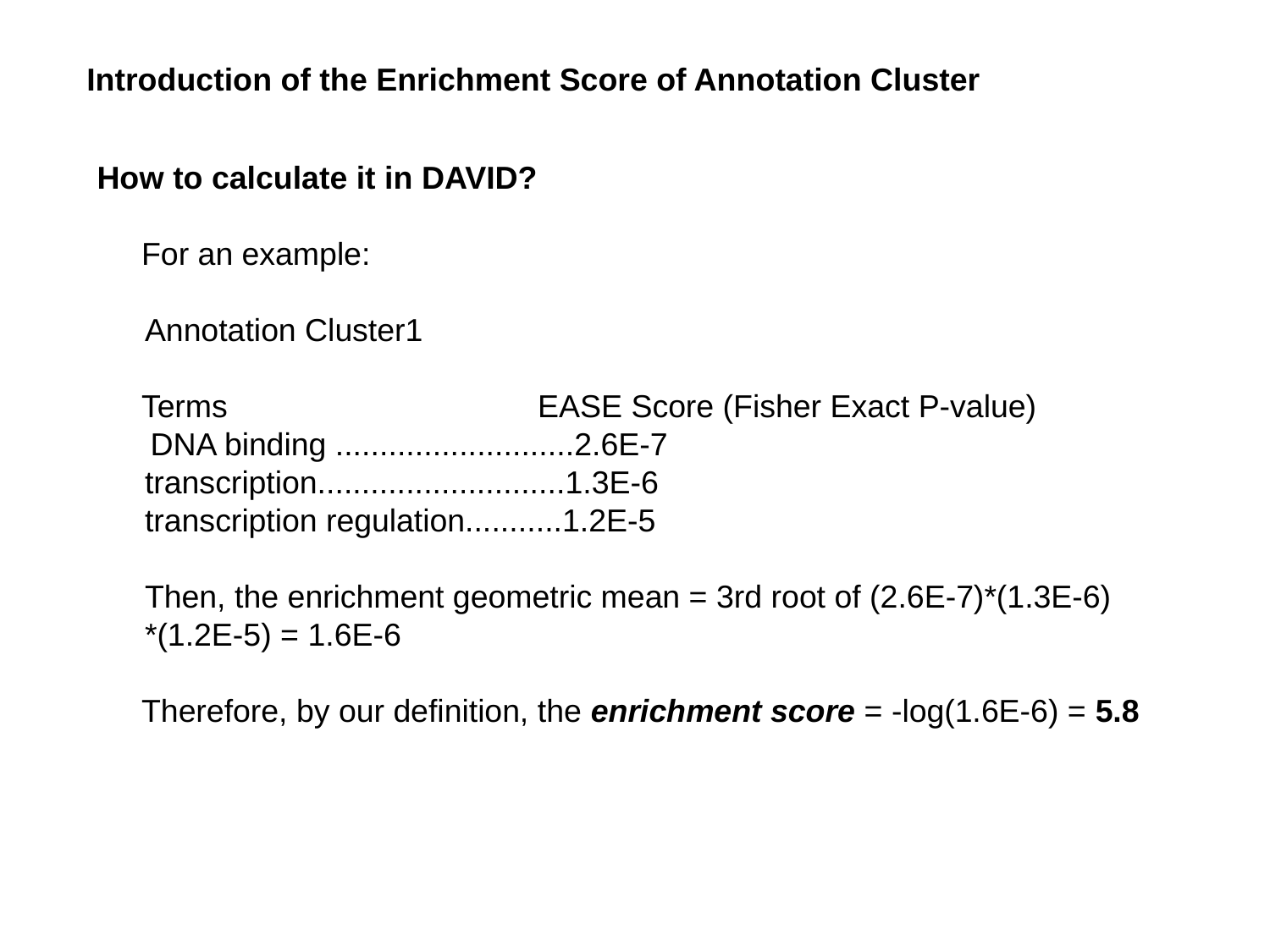

Introduction of the Enrichment Score of Annotation Cluster
How to calculate it in DAVID?
 For an example:
Annotation Cluster1
 Terms EASE Score (Fisher Exact P-value)
 DNA binding ...........................2.6E-7
transcription............................1.3E-6
transcription regulation...........1.2E-5
Then, the enrichment geometric mean = 3rd root of (2.6E-7)*(1.3E-6) *(1.2E-5) = 1.6E-6
 Therefore, by our definition, the enrichment score = -log(1.6E-6) = 5.8
