# Supplementary material for: The DAVID Gene Functional Classification Tool: a novel biological module-centric algorithm to functionally analyze large gene lists
Source: Genome Biol. 2007 Sep 4;8(9):R183. doi: 10.1186/gb-2007-8-9-r183 (PMC2375021; doi:10.1186/gb-2007-8-9-r183)
Supplement: Additional data file 7 — Fourteen annotation categories used in the DAVID Functional Classification Tool. [file gb-2007-8-9-r183-S7.doc]

|  | **Annotation Categories** | **Links & References** |
| --- | --- | --- |
| 1 | GO Biological Process | - Data extract from [LocusLink](http://www.ncbi.nlm.nih.gov/LocusLink/), [PIR iProClass](http://pir.georgetown.edu/iproclass/), [UniProt](http://www.pir.uniprot.org/)  and [GOA](http://www.ebi.ac.uk/GOA/) records. - Additional information regarding Gene Ontology can be found at [http://www.geneontology.org](http://www.geneontology.org/). |
| 2 | GO Molecular Function |
| 3 | GO Cellular Component |
| 4 | COG/KOG Ontology | <http://www.ncbi.nlm.nih.gov/COG/new/> |
| 5 | SMART Domains | <http://smart.embl-heidelberg.de/> |
| 6 | InterPro Domains | <http://www.ebi.ac.uk/interpro/> |
| 7 | KEGG Pathways | <http://www.genome.jp/kegg/> |
| 8 | CGAP BioCarta Pathways | <http://cgap.nci.nih.gov/Pathways/BioCarta_Pathways> |
| 9 | BBID Pathways | <http://bbid.grc.nia.nih.gov/> |
| 10 | UniProt Sequence Features | <http://www.pir.uniprot.org/> |
| 11 | Swiss-Prot Keywords | <http://www.pir.uniprot.org/> |
| 12 | PIR SuperFamily Names | <http://pir.georgetown.edu/iproclass/> |
| 13 | NIH Genetic Association DB | <http://geneticassociationdb.nih.gov/> |
| 14 | NCBI OMIM | <http://www.ncbi.nlm.nih.gov/entrez/query.fcgi?db=OMIM> |

**14 Annotation Categories Used in DAVID Functional Classification Tool**
