# Supplementary material for: The DAVID Gene Functional Classification Tool: a novel biological module-centric algorithm to functionally analyze large gene lists
Source: Genome Biol. 2007 Sep 4;8(9):R183. doi: 10.1186/gb-2007-8-9-r183 (PMC2375021; doi:10.1186/gb-2007-8-9-r183)
Supplement: Additional data file 8 — Graphical instruction and tutorial on how to use the DAVID Functional Classification Tool and the DAVID Functional Annotation Clustering Tool. [file gb-2007-8-9-r183-S8.doc]

**A Tutorial/An Example to Use the DAVID Functional Classification Tool**

Step 1: Start analysis


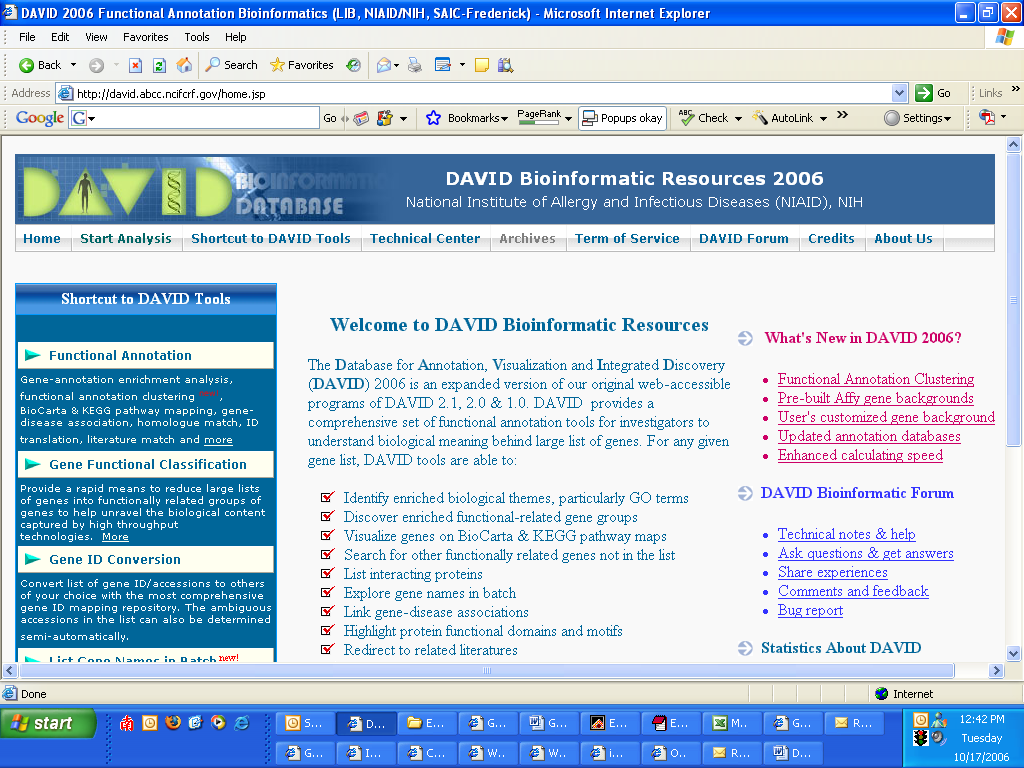


Step 2: Submit gene list or use built-in demo_lists


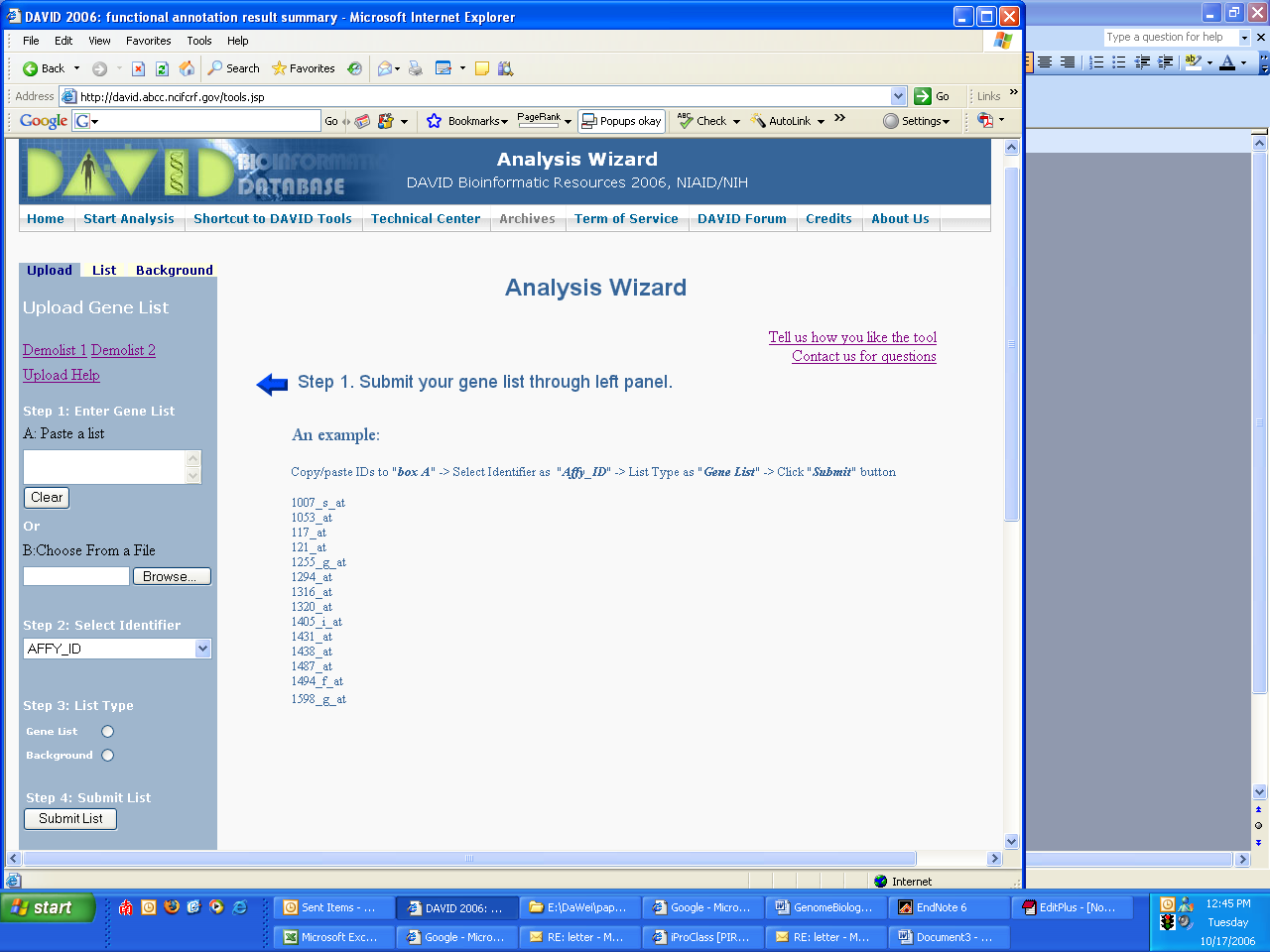


Step 3: Select the DAVID Gene Functional Classification Tool


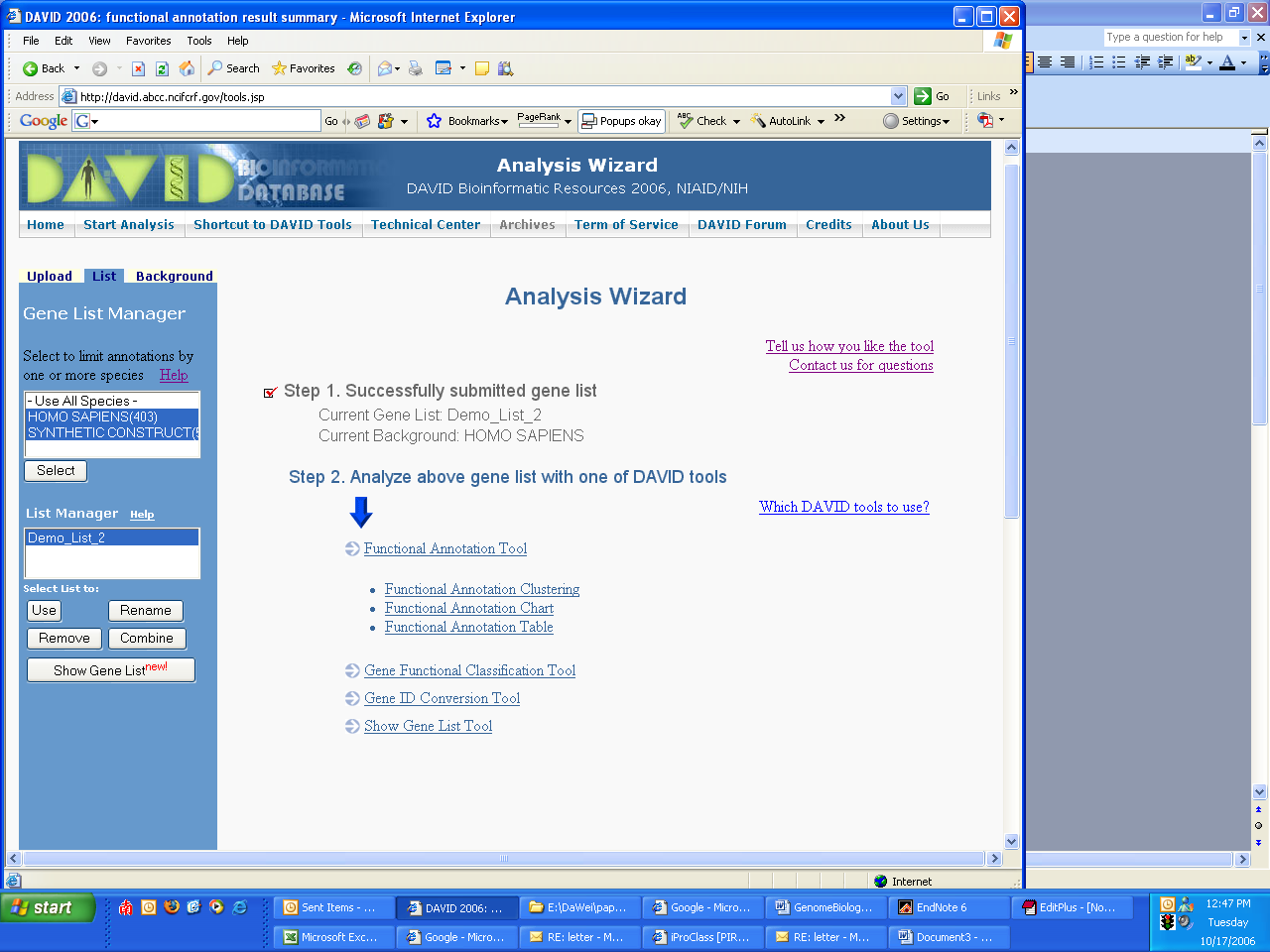


Step 4. View and explore results


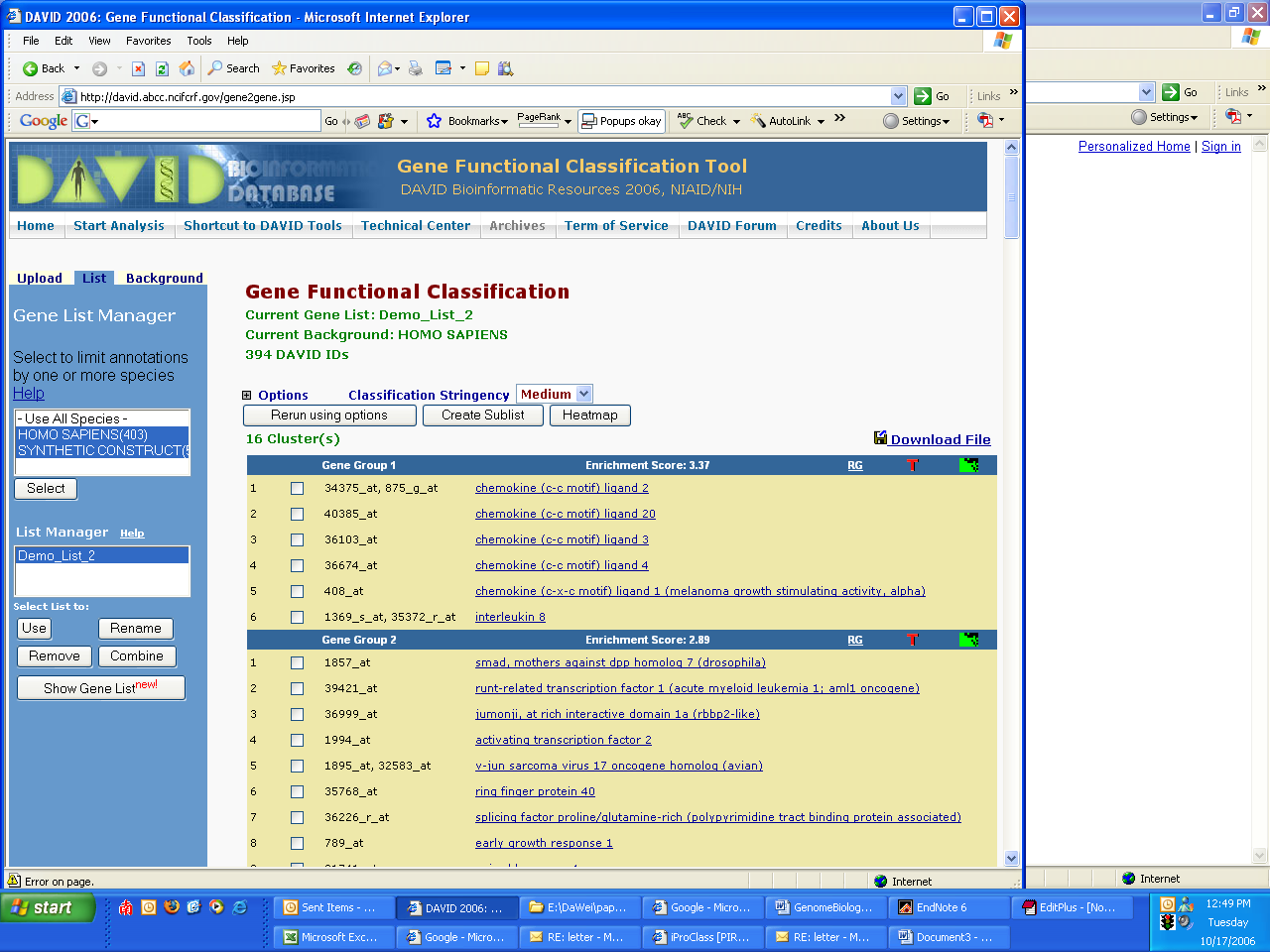


Finished

**A Tutorial/an Example to Use the DAVID Annotation Clustering Tool**

To repeat Step 1-3 above.

Or continue with Step 4 shown above

Step 5. Reuse the gene list by switching to other DAVID tools


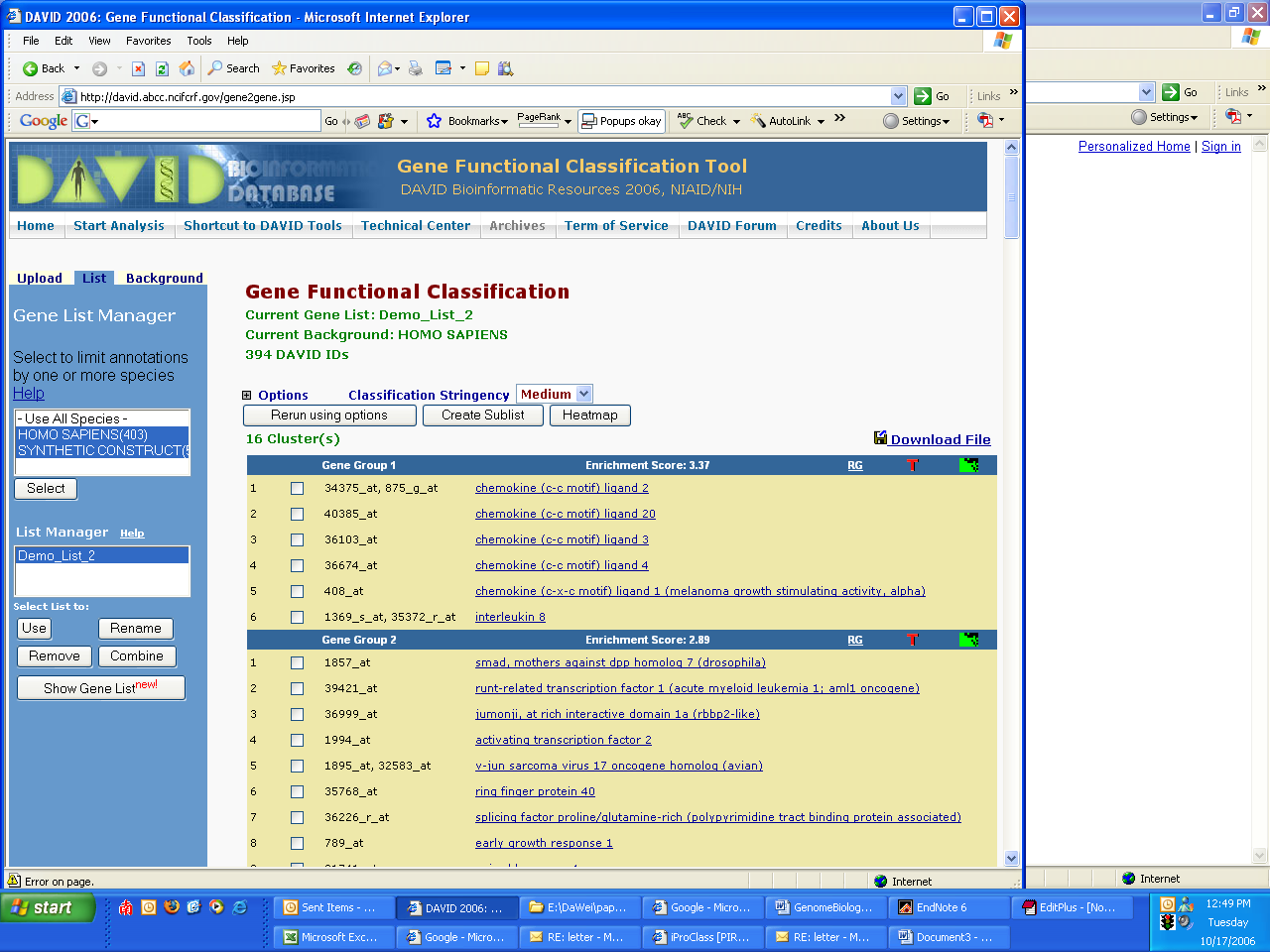


Step 6. Select the DAVID Functional Annotation Clustering


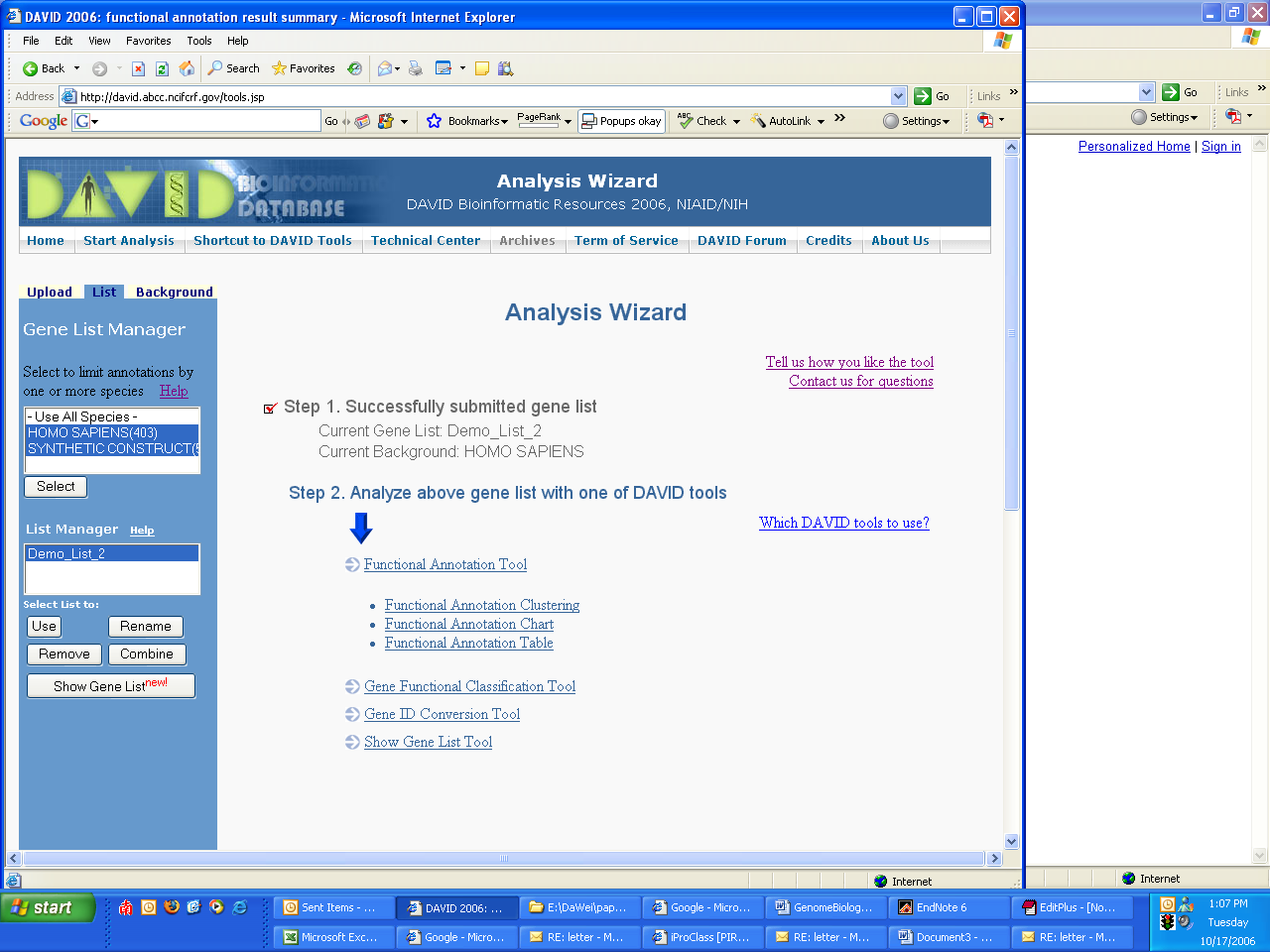


Step 7. Run the DAVID Functional Annotation Clustering


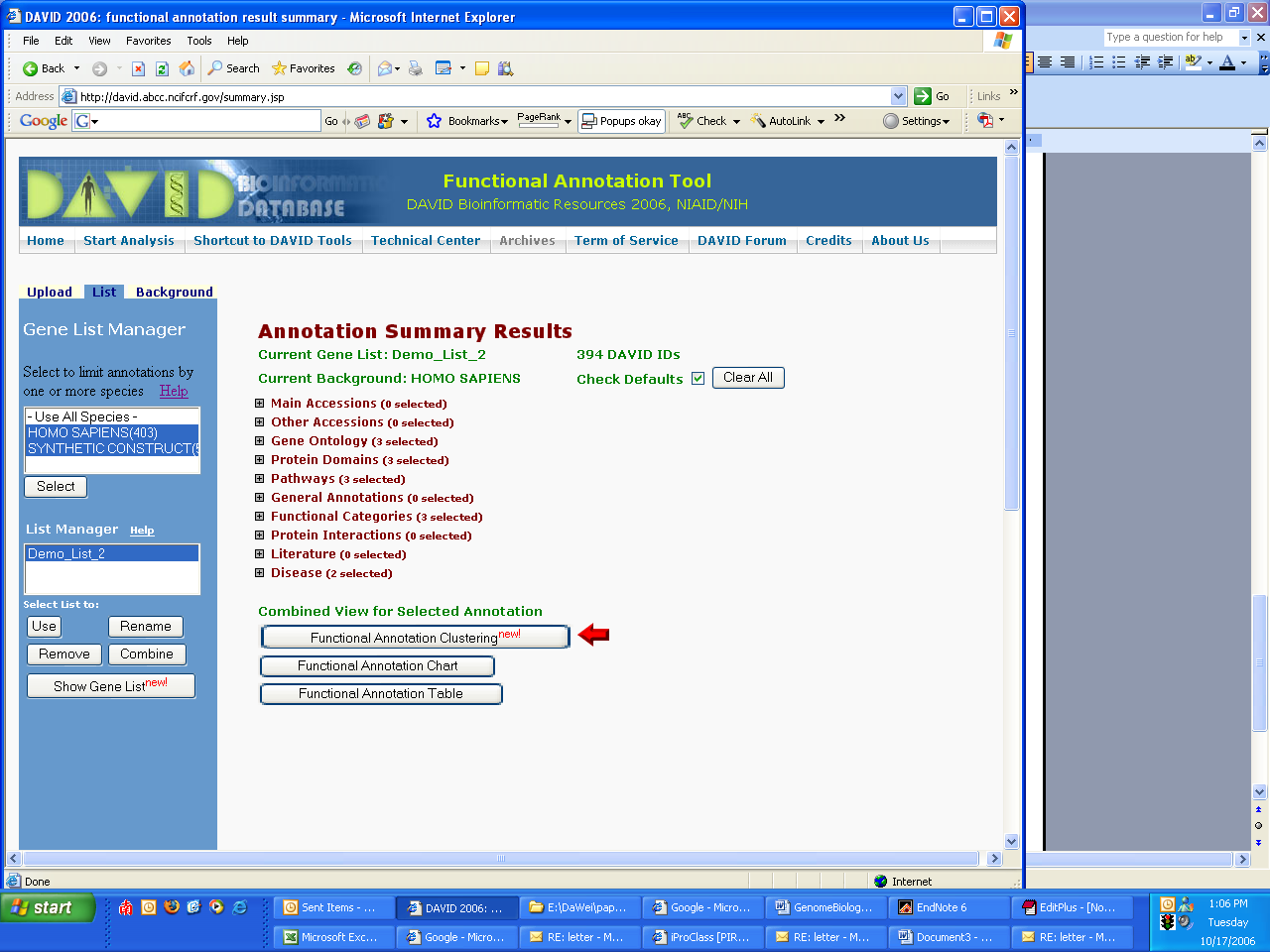


Step 8. View and explore results


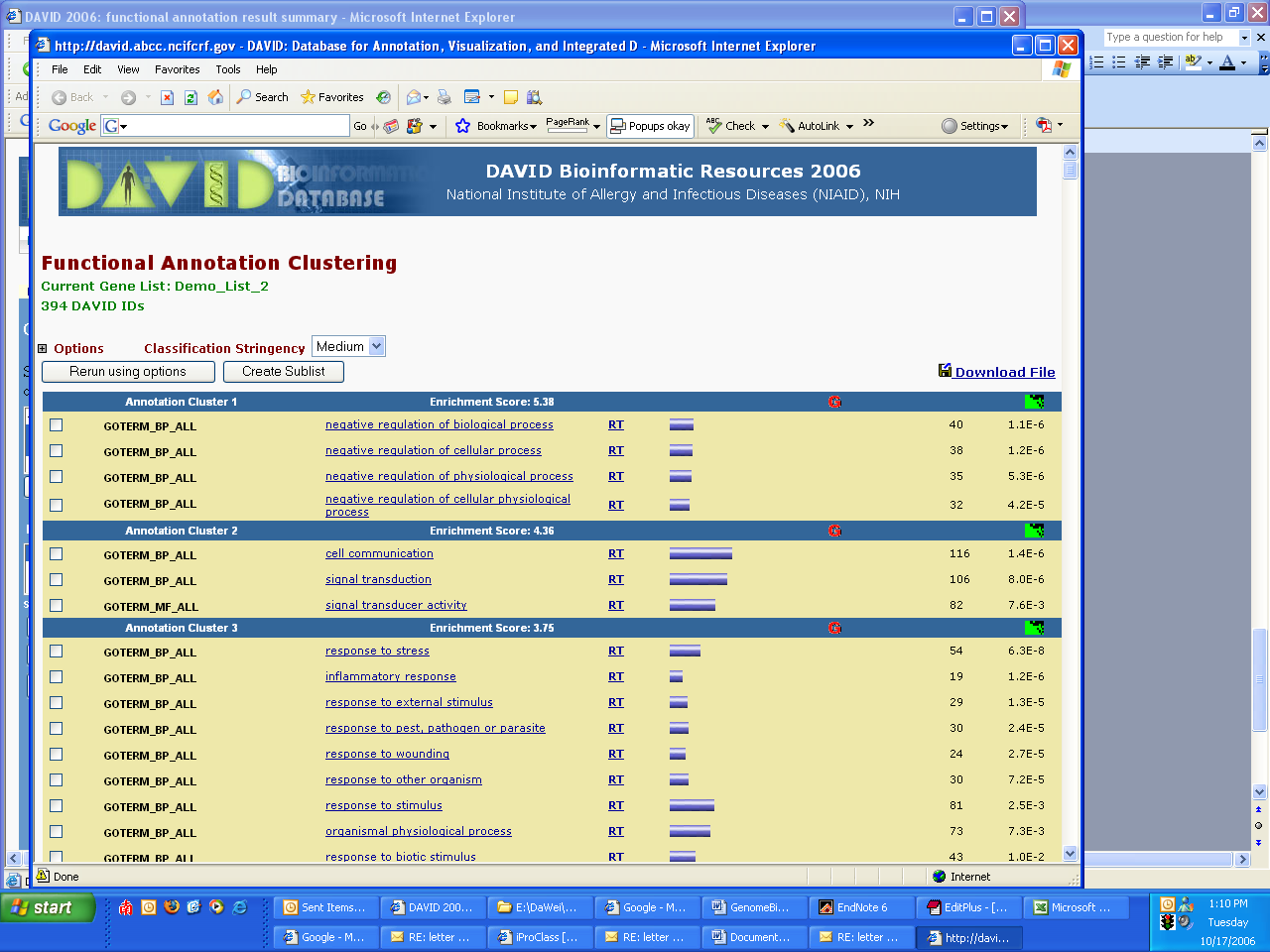


Finished
