# Supplementary material for: The DAVID Gene Functional Classification Tool: a novel biological module-centric algorithm to functionally analyze large gene lists
Source: Genome Biol. 2007 Sep 4;8(9):R183. doi: 10.1186/gb-2007-8-9-r183 (PMC2375021; doi:10.1186/gb-2007-8-9-r183)
Supplement: Additional data file 9 — (a) Related gene search for 'interleukin 8' in the scope of demo list 2. (b) Related term search for 'inflammatory response' in the scope of all annotations. (c) Related gene search for a group of genes, group 1 for demo list 2, identified by the DAVID Gene Functional Classification Tool. [file gb-2007-8-9-r183-S9.ppt]

## Slide 1
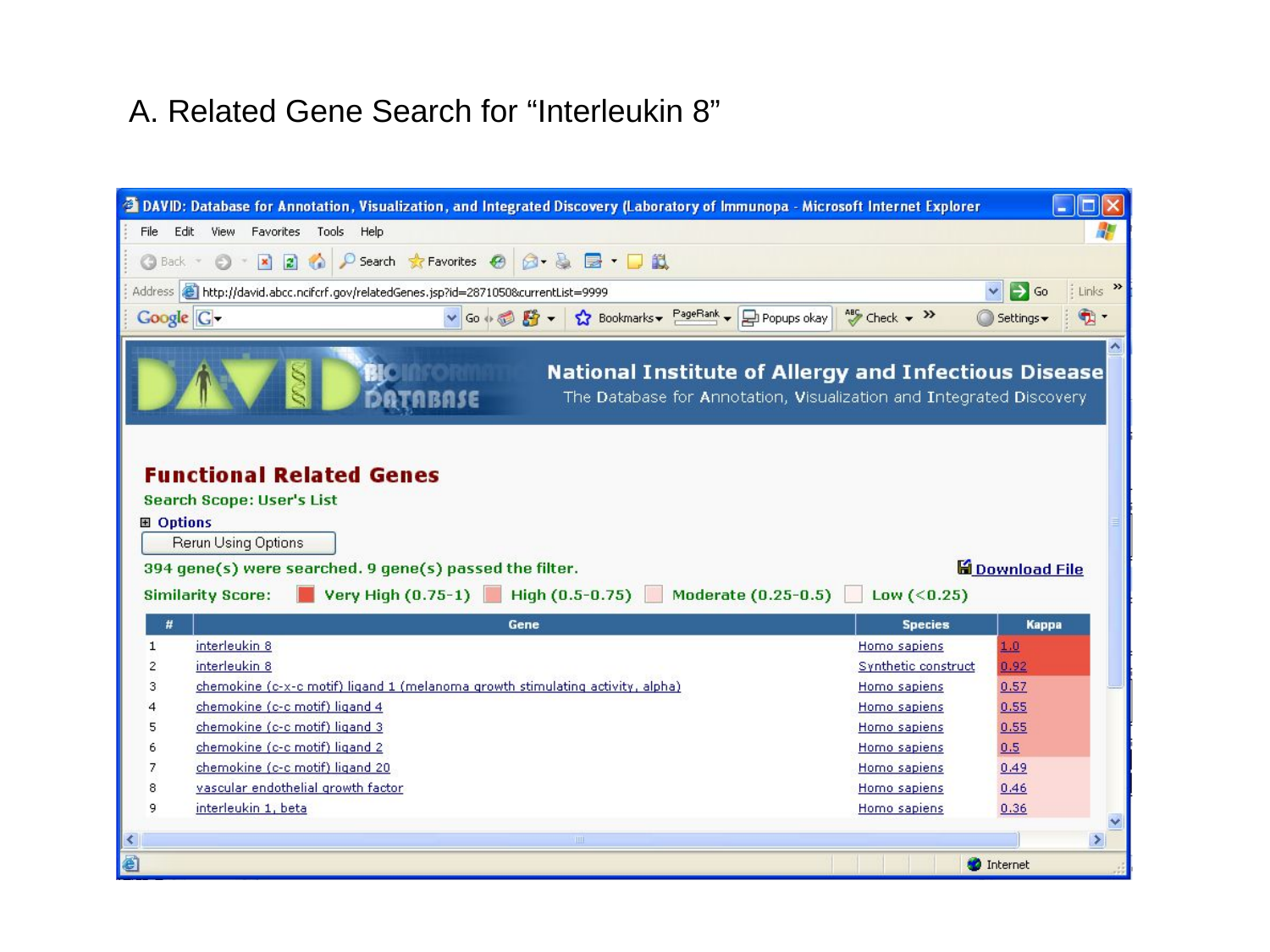

A. Related Gene Search for “Interleukin 8”

## Slide 2
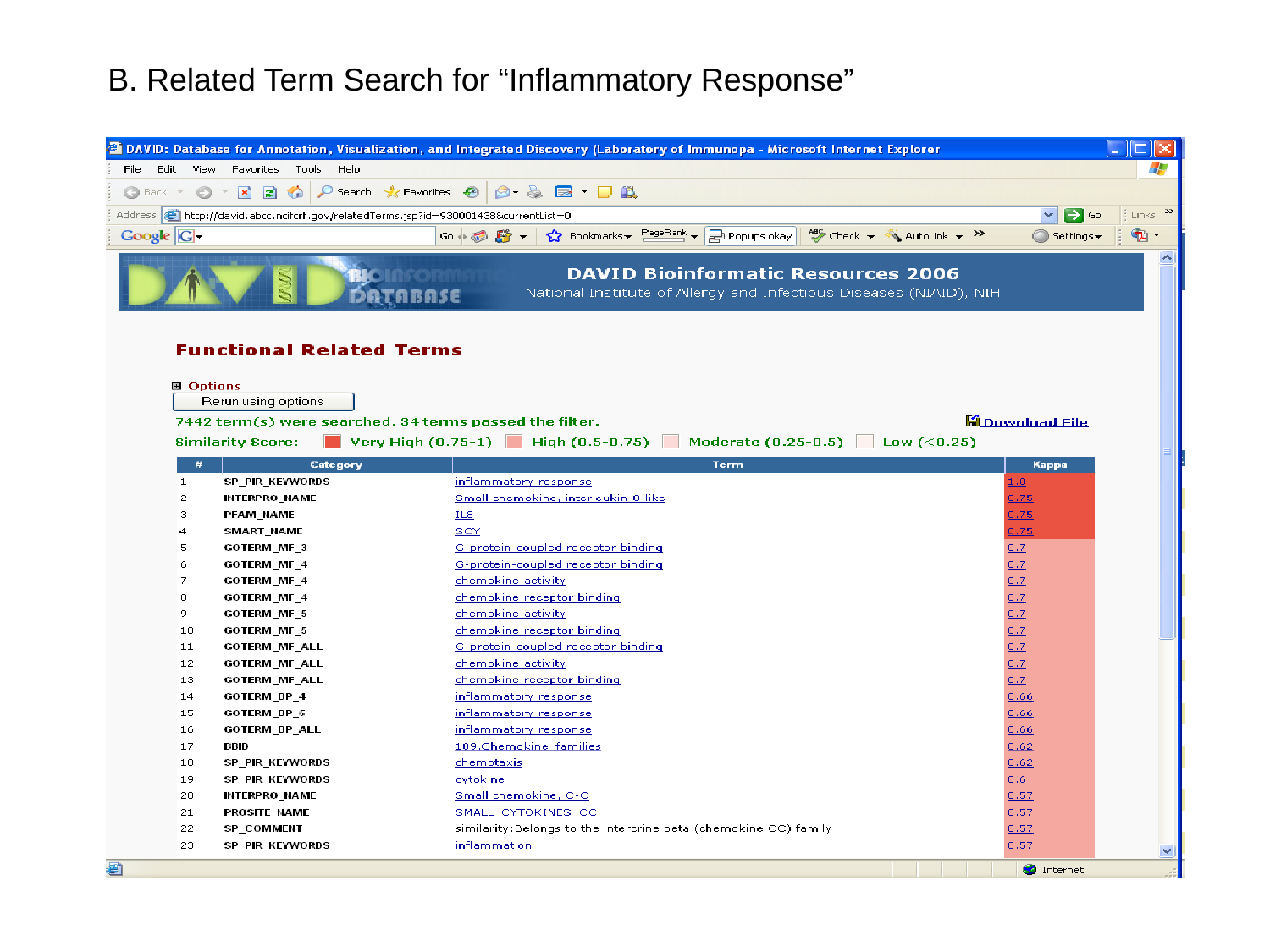

B. Related Term Search for “Inflammatory Response”

## Slide 3
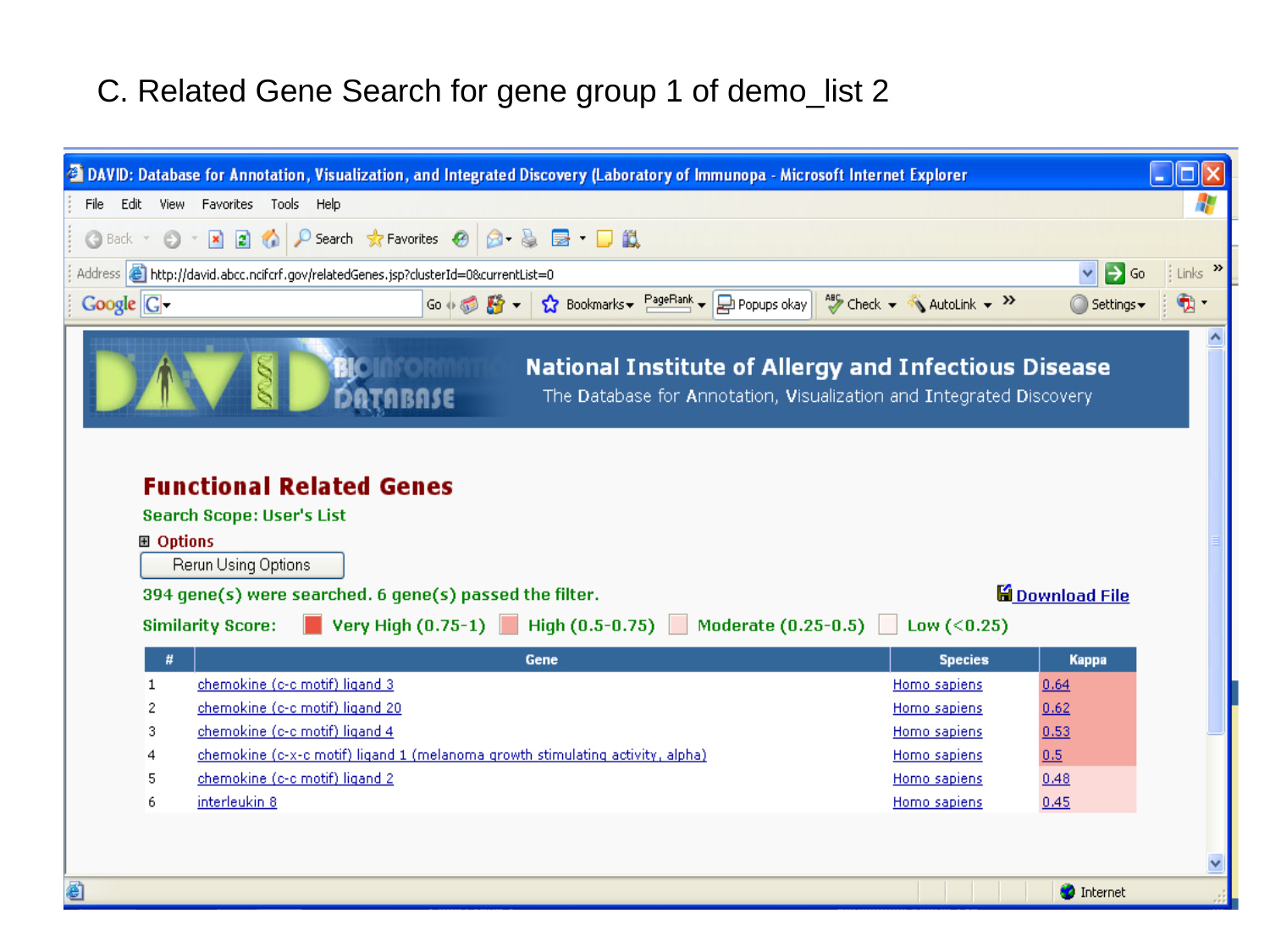

C. Related Gene Search for gene group 1 of demo_list 2
