# Supplementary material for: The DAVID Gene Functional Classification Tool: a novel biological module-centric algorithm to functionally analyze large gene lists
Source: Genome Biol. 2007 Sep 4;8(9):R183. doi: 10.1186/gb-2007-8-9-r183 (PMC2375021; doi:10.1186/gb-2007-8-9-r183)
Supplement: Additional data file 10 — (a) Significant kappa scores (≥0.35 based on randomization study in Figure 3) can be obtained only for gene-gene pairs with higher overlapped annotation terms (≥10). Thus, there is no reason to calculate kappa scores, in an attempt to save the calculating time for DAVID Functional Classification, for the large number of those gene-gene pairs with fewer annotation terms overlapped. A conservative default filter is 4. (b) Such a default filer (blue curve) has somewhat greater impact on the significant kappa scores in the higher end, compared to those in the lower end. However, it will skip a significant amount of kappa calculation of gene-gene pairs. [file gb-2007-8-9-r183-S10.ppt]

## Slide 1
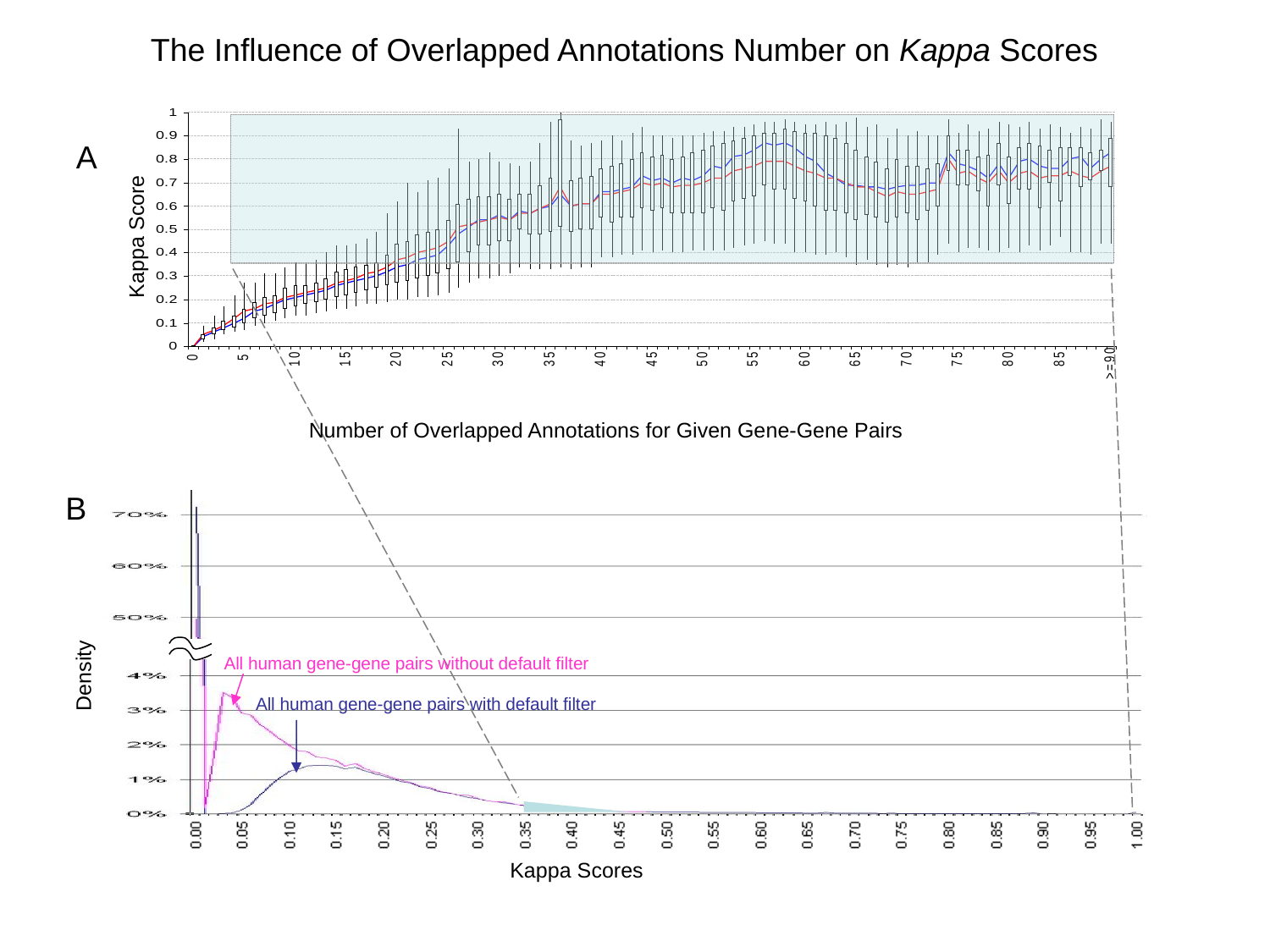

The Influence of Overlapped Annotations Number on Kappa Scores
Kappa Score
Density
Kappa Scores
A
Number of Overlapped Annotations for Given Gene-Gene Pairs
B
All human gene-gene pairs without default filter
All human gene-gene pairs with default filter
