# Supplementary material for: The DAVID Gene Functional Classification Tool: a novel biological module-centric algorithm to functionally analyze large gene lists
Source: Genome Biol. 2007 Sep 4;8(9):R183. doi: 10.1186/gb-2007-8-9-r183 (PMC2375021; doi:10.1186/gb-2007-8-9-r183)
Supplement: Additional data file 11 — The annotation data contents contain many more 0 s than 1 s. The test shows that kappa statistics is able to detect 1-1 relationships, which are the key biological co-occurrences that we desire to measure. [file gb-2007-8-9-r183-S11.doc]

**Effect of Kappa Statistics on Biased Annotation Data**

**1. What does annotation data matrix look like?**

**Matrix of genes vs. terms**

|  | **term 1** | **term 2** | **term 3** | **term 4** | **term 5** | **……..** | **……..** | **……..** | **term 20,000** |
| --- | --- | --- | --- | --- | --- | --- | --- | --- | --- |
| **Gene A** | **1** | **1** | **0** |  | **0** | **0** | **0** | **0** | **0** |
| **Gene B** | **1** | **0** | **0** | **1** | **0** | **0** | **0** | **0** | **0** |
| **Gene C** | **1** | **0** | **0** | **1** | **0** | **0** | **0** | **0** | **0** |
| **Gene D** | **0** | **0** | **1** | **0** | **0** | **0** | **0** | **0** | **0** |

**Problem: There are dominate numbers of 0s than 1s in the matrix.**

**2. Does the biased 2x2 contingency table fail our data mining goal?**

| **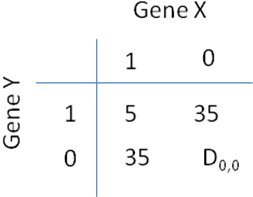** | **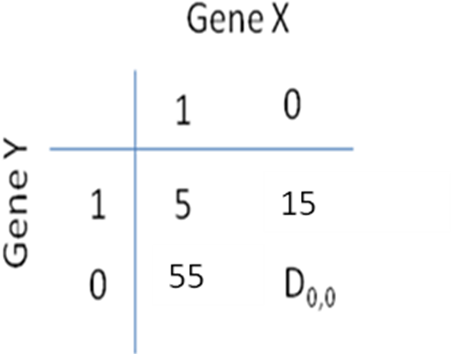** |
| --- | --- |
| **Table 1** | **Table 2** |

**Assume Gene X and Y have average amount of terms (e.g. 75 terms in total). They are not agreed with each other for most of cases, e.g. For table 1, 35 and 35 represent numbers of disagreed terms between gene X and Y; 5 represents the agreed terms. In general, Gene X and Y have much disagreement than agreement in both table 1 and table 2. Now, when D_0,0_ is really large (e.g. ~ 5,000) due to the nature of raw annotation data matrix (see the first table above), does this bias will alter the measurement capability of Kappa Statistics? To answer the question, we calculate Kappa values for both table 1 & 2 with D_0,0_ being continuously increased from 100 to 7000 . Figure below indicates that Kappa values are fairly stable and correct regardless the increased D_0,0_ numbers. In another word, the unrelated nature of X and Y are measured correctly (small kappa values) despite the big D_0,0_.**

**
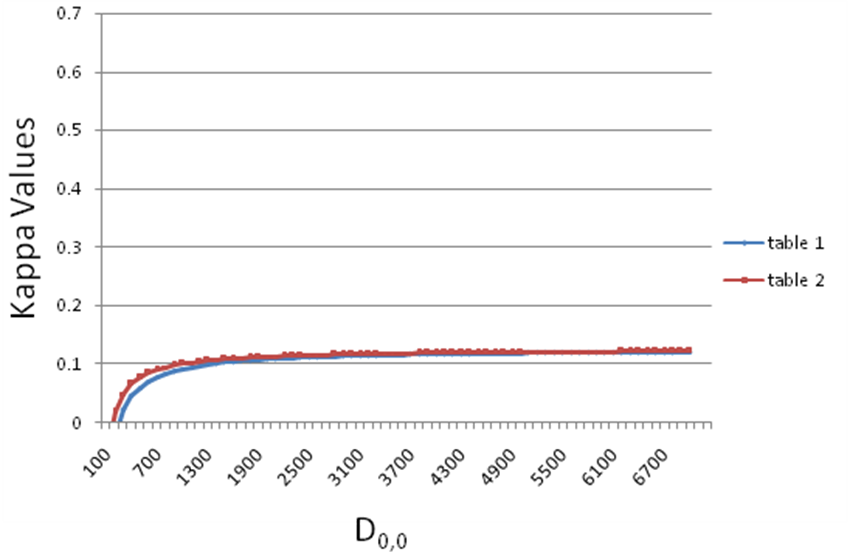
**

**3. Then, what are the driving** factors for Kappa values in this case?

| **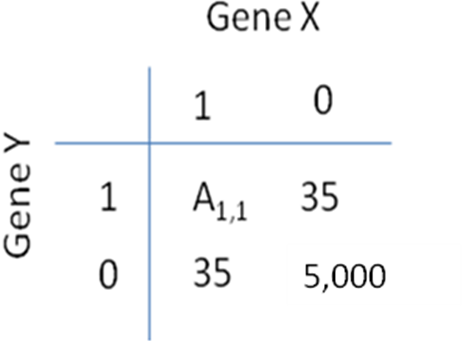** | **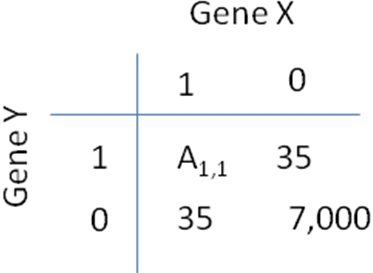** | **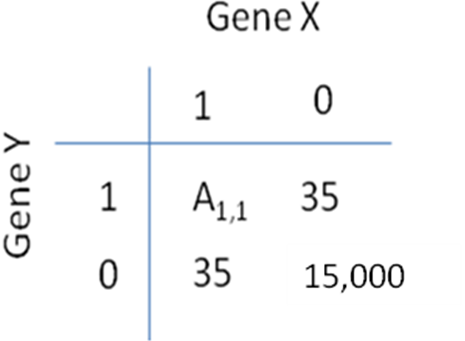** |
| --- | --- | --- |

**Assume D_0,0_ are constant values of 5000, 7000 or 1500, which are very close to the true range of the real annotation data set. Then, we calculate three curves for Kappa values for 5000, 7000 and 15000 respectively with A _1,1_  being continuously increased from 0 to 70. The figure below indicates that Kappa values are overlapped despite different D_0,0_ values. A _1,1_  is one the major factors to control Kappa values because it s increases are correlated with the increases of Kappa values. Because A _1,1_  is the most interesting biological factors we desire to measure, the Kappa statistics meets what we need for annotation data mining.**

**
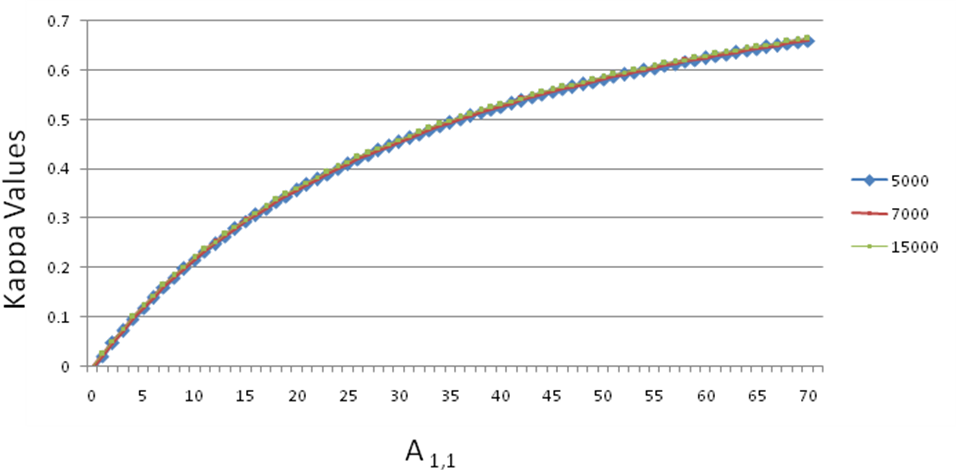
**

**4. Discussion**

**A biased 2x2 contingency table might alter the Kappa values in a certain way. It is a problem only when Kappa values are compared between one from a balanced table and the other from biased table. Since all our Kappa values in the annotation data mining have similar biased tables, the Kappa values are highly comparable to each other on the same floor. The bias-adjusted Kappa (BAK)*, which re-emphasizes the contributions of D_0,0_ , does not favor our situation (data not shown). The figures presented above, combined with other evidences in the manuscript, suggests that Kappa statistics is a reliable measurement of the true gene-term relationships in our algorithm.**

**** Byrt T. Bishop J. Carlin JB. Bias, prevalence and kappa. Journal of Clinical Epidemiology. 46(5):423-9, 1993 May.***
