# Supplementary material for: The DAVID Gene Functional Classification Tool: a novel biological module-centric algorithm to functionally analyze large gene lists
Source: Genome Biol. 2007 Sep 4;8(9):R183. doi: 10.1186/gb-2007-8-9-r183 (PMC2375021; doi:10.1186/gb-2007-8-9-r183)
Supplement: Additional data file 12 — The examples suggest that the 'flat' matrix strategy, along with kappa statistics, allows for the quantitative measurement of gene-gene and term-term relationships based on global annotation profiles. All levels of annotation are important to measurement contribution. [file gb-2007-8-9-r183-S12.doc]

# A hypothetical example to illustrate the measurement of gene-gene relationships by Kappa Statistics

**Step 1. A structured GO information is converted into a flat matrix.**

**
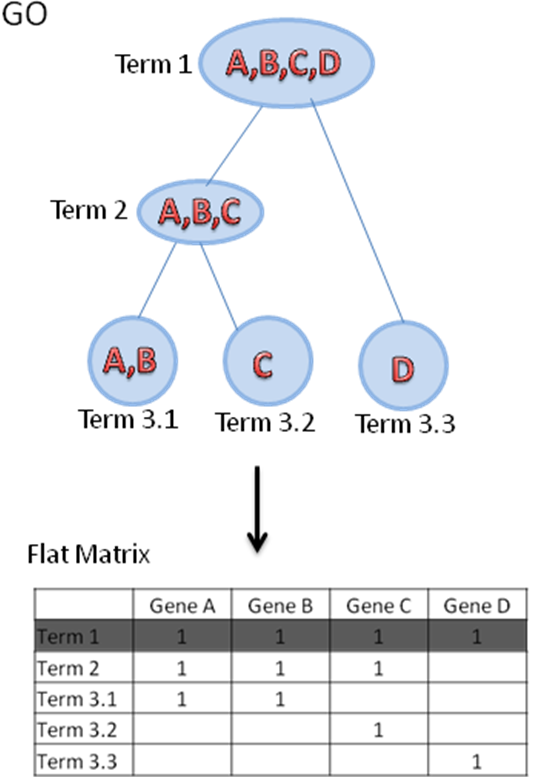
**

**Step 2. Measuring gene-gene relationships based on the information with and without high level GO term 1.**

**
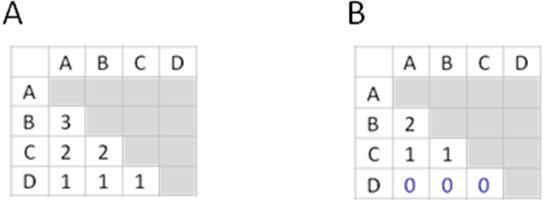
**

**Based on the ‘flat’ matrix in step 1, # of co-occurrence terms can be counted. The numbers roughly represent the strength of relevance of gene- gene because they are the key factors driving kappa values. A) The measurements with all GO terms including highest level term 1 (non-specific) can distinguish the relative degree of relationships among gene A, B, C, D. For example, A-B with 3 shows a stronger relationship than A-D with 1. The relationship measurement based on the flat matrix is consistent with the information in theGO tree that A-B carries through all levels of GO terms, and A-D does not. B) Now we only focus on specific terms so that high level term 1 is excluded (equivalent to weight = 0). After re-constructing of the flat matrix (remove the first highlighted row in flat matrix), the conclusion, that A-B (2) is stronger than A-D (0), is still similar to previous one. However, the measurement power decreases from 3 to 2. Without the higher level of GO, the relationship of A-D cannot be detected. All together, it indicates that considering non-structured terms in the flat matrix will enhance the measurement power of gene-gene relationship without altering the key relationship trends. Due to many intermediate terms (or parent terms like term 2) in the middle between the specific and non-specific terms will always give different "votes" toward the specific terms, the strategy of global measurements in the "flat" matrix can fairly represent the true global profiles of the internal biological connections. Importantly, this natural power of favorite specific terms is also probably true for those annotations without GO-like structures.**

**Step 3. Measuring term-term relationships based on the information with and without high level GO term 1.**

**
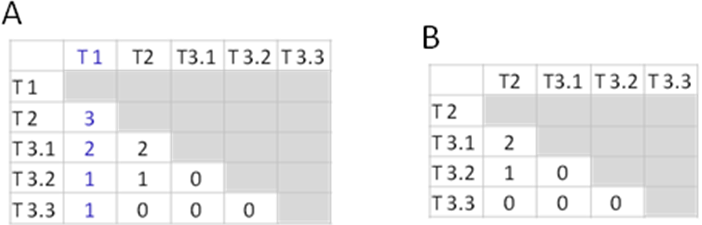
**

**Based on the ‘flat’ matrix in step 1, # of co-occurrence genes can be counted. The numbers roughly represent the strength of relevance of term-term because they are the key factors driving kappa values. A) The measurements with all GO terms including high level term 1 (non-specific) indicate the relatively stronger relationships of T1-T2 (3), T2-T3.1(2) and T1-T3.1(2), which represents the true structure of the GO hierarchy. Sometimes, non-specific terms can have considerable relationship with specific terms, e.g. T1-T3.1. For most cases, it does not, e.g. T1-T3.2 and T1-T3.3. The advancement of the algorithm is that the relationships across different levels is dynamically determined based on the biology rather than a static threshold for all situations. B) Now we only focus on specific terms so that high level term 1 is excluded (equivalent to weight = 0). The measurements have not changed except for the T1 column is removed. Thus, T1 does not participate in any relationships and all of the remaining relationships stay the same. All together, it indicates that the flat matrix strategy allows the relationship to be quantitatively measured. The non-specific terms will not alter the measurement for term-term relationships.**
