# Supplementary material for: The DAVID Gene Functional Classification Tool: a novel biological module-centric algorithm to functionally analyze large gene lists
Source: Genome Biol. 2007 Sep 4;8(9):R183. doi: 10.1186/gb-2007-8-9-r183 (PMC2375021; doi:10.1186/gb-2007-8-9-r183)
Supplement: Additional data file 13 — The example provides a step-by-step demonstration of the clustering algorithm, thereby showing how the members are grouped together, how the number of total groups are determined, and how fuzziness can occur. [file gb-2007-8-9-r183-S13.doc]

**A Hypothetical Example of the DAVID Clustering**

**Raw Data**

Profile of genes vs. terms

|  | t1 | t2 | t3 | t4 | t5 | t6 | t7 | t8 | t9 | t10 | t11 | t12 | t13 | t14 | t15 |
| --- | --- | --- | --- | --- | --- | --- | --- | --- | --- | --- | --- | --- | --- | --- | --- |
| gene a | 1 | 1 | 1 | 1 | 1 | 1 | 1 | 1 | 1 | 1 | 0 | 0 | 0 | 0 | 0 |
| gene b | 1 | 1 | 1 | 1 | 1 | 1 | 1 | 1 | 1 | 1 | 0 | 0 | 0 | 0 | 0 |
| gene c | 1 | 1 | 1 | 1 | 1 | 1 | 1 | 1 | 1 | 1 | 0 | 0 | 0 | 0 | 0 |
| gene d | 0 | 0 | 0 | 0 | 1 | 1 | 1 | 1 | 1 | 1 | 1 | 0 | 0 | 0 | 0 |
| gene e | 0 | 0 | 0 | 0 | 0 | 1 | 1 | 1 | 1 | 1 | 1 | 1 | 1 | 1 | 1 |
| gene f | 0 | 0 | 0 | 0 | 0 | 1 | 1 | 1 | 1 | 1 | 1 | 1 | 1 | 1 | 1 |
| gene g | 0 | 0 | 0 | 0 | 0 | 1 | 1 | 1 | 1 | 1 | 1 | 1 | 1 | 1 | 1 |
| gene h | 0 | 0 | 0 | 1 | 0 | 0 | 0 | 1 | 0 | 0 | 0 | 1 | 0 | 0 | 0 |

Visually, gene a, b, and c share similar profile of terms. Gene e, f, and g have common profile. Gene d could be in either groups. Gene h (yellow) is an outlier not closely associating with any of the groups. Therefore, there are two major gene groups (blue and red), and gene d (green) could belong to either groups.

**Goal**

Based on the given profile of terms , to systematically determine the number of potential gene groups and also classify genes into each groups.

**DAVID Clustering: A Heuristic Multiple Linkage Fuzzy Clustering Procedure**

Step 1: Measure the relationships of all gene-gene pairs with Kappa statistics (figure 2)(or any distance measurement). A heuristic threshold of kappa value is 0.35 (i.e. 'Kappa similarity' threshold in DAVID interface). Any values above it (in red) are considered as significant relationships.

|  | **a** | **b** | **c** | **d** | **e** | **f** | **g** | **h** |
| --- | --- | --- | --- | --- | --- | --- | --- | --- |
| **a** |  | 1 | 1 | 0.35 | -0.50 | -0.50 | -0.50 | 0.00 |
| **b** | 1 |  | 1 | 0.35 | -0.50 | -0.50 | -0.50 | 0.00 |
| **c** | 1 | 1 |  | 0.35 | -0.50 | -0.50 | -0.50 | 0.00 |
| **d** | 0.35 | 0.35 | 0.35 |  | 0.35 | 0.35 | 0.35 | -0.11 |
| **e** | -0.50 | -0.50 | -0.50 | 0.35 |  | 1 | 1 | 0.00 |
| **f** | -0.50 | -0.50 | -0.50 | 0.35 | 1 |  | 1 | 0.00 |
| **g** | -0.50 | -0.50 | -0.50 | 0.35 | 1 | 1 |  | 0.00 |
| **h** | 0.00 | 0.00 | 0.00 | -0.11 | 0.00 | 0.00 | 0.00 |  |

Step 2: Create qualified initial seeding groups: Each gene could form a initial seeding group (initial seeds) as long as it has close relationships (e.g. kappa >=0.35) with more than > 2 other members (i.e. 'initial group membership' threshold in DAVID interface) . In order to control the quality of the seeding groups, the qualified seeding groups (qualified seeds) need to meet the second condition, i.e. majority (>50%) of members in the seed should have close relationships (e.g. kappa >= 0.35) each other. For example, ' d->a b c e f g' is not qualified because too many (>50%) gene pairs like a-e, a-f, a-g, b-e, b-f, b-g, etc. do not show good relationships (e.g. kappa <0.3). Therefore, ' d->a b c e f g' and 'h->' do not meet above two heuristic conditions.

| **Initial seeds** | **# of membership** | **% of tighter relationships (> 0.35) within a seed** | **Qualified seeds** |
| --- | --- | --- | --- |
| a-> b c d  b->a c d  c->a b d  d->a b c e f g  e->d fg  f-> d e g  g-> d e f  h-> | >2  >2  >2  >2  >2  >2  >2  <2 | >50%  >50%  >50%  <50%  >50%  >50%  >50% | 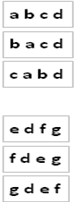 |

Step3: Iteratively merging above qualified seeds: Any two seeds have chances to be merged if they share majority (e.g. >50%) of members (i.e. "Multiple Linkage' threshold in DAVID interface). For example, 'abcd' and 'bacd' are merged due to sharing 100% members in loop No. 1. Merging keep going until all groups are stable, i.e. no any two seeds and intermediate groups share more than >50% members. The dash lines represent the stop points to start next new loop.


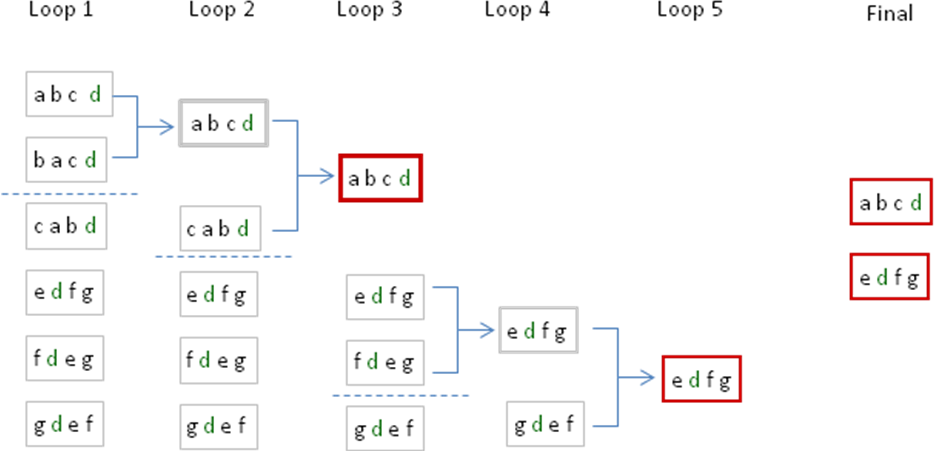


**Result**

Two gene groups are discovered, 'abcd' and 'efgd'. Gene d (in green) is assigned to two groups respectively. Gene h is an outlier. Results are consistent with human visual judgment at beginning.

**Key Points**

- Number of total groups is dynamically determined based on the given conditions.
- Fuzziness: one member could be in more than one groups, e.g. gene d is in both groups.
- Outliers are filtered out, e.g. gene h.
- Method could be expanded to other applications, such as microarray expression clustering.
